# Deciphering the function of Com_YlbF domain-containing proteins in *Staphylococcus aureus*

**DOI:** 10.1128/jb.00061-25

**Published:** 2025-08-18

**Authors:** Zayda-Lorena Corredor-Rozo, Ricaurte Marquez-Ortiz, Myriam L. Velandia-Romero, Deisy Abril, Johana Madroñero, Luisa F. Prada, Natasha Vanegas-Gomez, Begoña García, Maite Echeverz, María-Angelica Calderón-Peláez, Jacqueline Chaparro‑Olaya, Liliana Morales, Carlos Nieto‑Clavijo, Javier Escobar-Perez

**Affiliations:** 1 Bacterial Molecular Genetics Laboratory, Vicerrectoría de Investigaciones, Universidad El Bosque 28009, Bogotá, Colombia; 2 Grupo de Virología, Vicerrectoría de Investigaciones, Universidad El Bosque 28009, Bogotá, Colombia; 3 Australian Institute for Microbiology and Infection, School of Life Sciences, Faculty of Science, University of Technology Sydney 1994, Sydney, New South Wales, Australia; 4 Laboratory of Microbial Pathogenesis, Navarrabiomed-Universidad Pública de Navarra (UPNA)-Hospital Universitario de Navarra (HUN), IdiSNA 16756, Pamplona, Spain; 5 Laboratorio de Parasitología Molecular, Vicerrectoría de Investigaciones, Universidad El Bosque 28009, Bogotá, Colombia; University of Illinois Chicago, Chicago, Illinois, USA

**Keywords:** *Staphylococcus aureus*, Com_YlbF domain proteins, biofilm, virulence, RNase-Y

## Abstract

The Com_YlbF domain-containing proteins inhibit sporulation, competition, and biofilm formation by affecting the activity of ribonuclease RNase-Y in *Bacillus subtilis*. Similar Com_YlbF proteins are found in *Staphylococcus aureus*, but their function is yet to be determined. This study investigates the role of Com_YlbF domain-containing proteins (Qrp/YheA, YmcA, and YlbF) in *S. aureus* by evaluating the impact of *qrp/yheA*, *ymcA*, and *ylbF* gene deletion on biofilm formation, PIA/PNAG production, and hemolytic capacity. It was found that deletion of the three *qrp/yheA*, *ymcA*, and *ylbF* genes in *S. aureus* produced a decrease in biofilm formation, a slight decrease in PIA/PNAG production, and a decrease in its hemolytic capacity. Global transcriptional analysis in the mutant strain showed dysregulation of several genes associated with pathogenesis, most notably a decreased expression of the *agrA* gene (quorum sensing system), the *delta*, *alpha*, and *gamma* hemolysins, as well as the *sdrC* genes and *eap/map* (adhesion proteins) genes. It also showed an attenuation of virulence, manifesting as enhanced survival among *Galleria mellonella* larvae and BALB/c mice, possibly associated due to disrupted RNase-Y activity when the *qrp/yheA*, *ymcA*, and *ylbF* are deleted. In conclusion, this work supports the hypothesis that this new family of proteins containing a Com_YlbF domain is involved in the regulation of genes related to biofilm formation, hemolysis, and virulence in *S. aureus* through a change in RNase-Y activity.

**IMPORTANCE:**

The Com_YlbF proteins are a group of small proteins identified in some Gram-positive bacteria whose function has not been described, but whose deletion has been related to pleiotropic effects in *Bacillus subtilis* (reduction of biofilm ability, sporulation, and competence). However, the role of these proteins in *S. aureus* has been little studied. This study shows the importance of the Com_YlbF protein family in *S. aureus* involved in biofilm formation and hemolysis. The phenotypes could be explained by an alteration of the enzymatic activity of the endoribonuclease RNase-Y, resulting in reduced secretion of virulence factors. These findings represent a significant step forward in understanding the modulation of pathogenesis and virulence in *S. aureus*.

## INTRODUCTION

*Staphylococcus aureus* can cause a wide range of infections in humans, from superficial skin infections to severe and potentially life-threatening infections, such as sepsis, bacteremia, pneumonia, infective endocarditis, and osteomyelitis (1, 2). The ability of this bacterium to cause infection is due to the production of dozens of virulence factors, such as hemolysins, leukocidins, modulins, proteases, surface-associated protein adhesins, exotoxins, superantigens, DNases, exopolysaccharides, host immune evasion molecules, and immune modulators (3, 4). In addition, *S. aureus* forms biofilms on indwelling medical devices or implanted materials, leading to chronic and recurrent infections that are challenging to treat (5). The expression of these virulence factors is tightly regulated by a complex and dynamic circuit that rapidly responds to changing environmental conditions.

YlbF, YmcA, and more recently YheA (also known as Qrp, glutamine-rich protein) are a group of small proteins (10 to 15 kDa) that contain Com_YlbF domain spanning almost the entire protein and share 31%, 40%, and 47% identity, respectively, with *Bacillus subtilis* homologs (6, 7). Their function in *S. aureus* is still unknown. *In silico* analysis shows a wide distribution of these proteins in Gram-positive bacteria, suggesting their role in conserved biological processes. YmcA and YlbF have been characterized genetically and biochemically in *Bacillus subtilis*, another Gram-positive bacterium, where their deletion results in significant deficiencies in sporulation, competency, and biofilm formation (7, 8). In *B. subtilis*, YmcA and YlbF form a stable ternary complex with the YaaT protein (named Y-complex), the latter lacking the Com_YlbF domain required for spore and biofilm formation (9). The Y-complex modulates the function of two key regulatory transcriptional proteins: Spo0A and SinR. This includes increasing the phosphorylated-active form of Spo0A (9) and destabilizing SinR mRNA through cleavage by the endoribonuclease RNase-Y (9–12). Notably, the Y-complex physically interacts with RNase Y and is necessary for the processing and regulation of many mRNA transcripts (11).

Information on the Com_YlbF domain-harboring protein of *S. aureus* is very limited. A study performed by Deloughery et al. (11) using end-enrichment RNA sequencing assay (Rend-seq) showed that deletion of the *ylbF* gene altered RNase-Y mRNA processing of the *cggR(gapR)-gapA* operon and the riboswitches *SerS* and *ValS* (11). We also demonstrated that *S. aureus* expresses another Com_YlbF domain-containing protein, Qrp/YheA, which shares a highly conserved 3D structure with YmcA and YlbF, and contains a putative conserved motif (QQKQMQ) located in the central region of the Com_YlbF domain, which could be involved in its function (6). Here, we provide evidence that Qrp/YheA, YmcA, and YlbF proteins could modulate virulence and biofilm formation in *S. aureus* through post-transcriptional regulation mediated by RNase-Y activity (13, 14).

## RESULTS

### The Qrp/YheA, YmcA, and YlbF proteins could participate in the transcription of genes associated with virulence and biofilms

**Fig 1:**
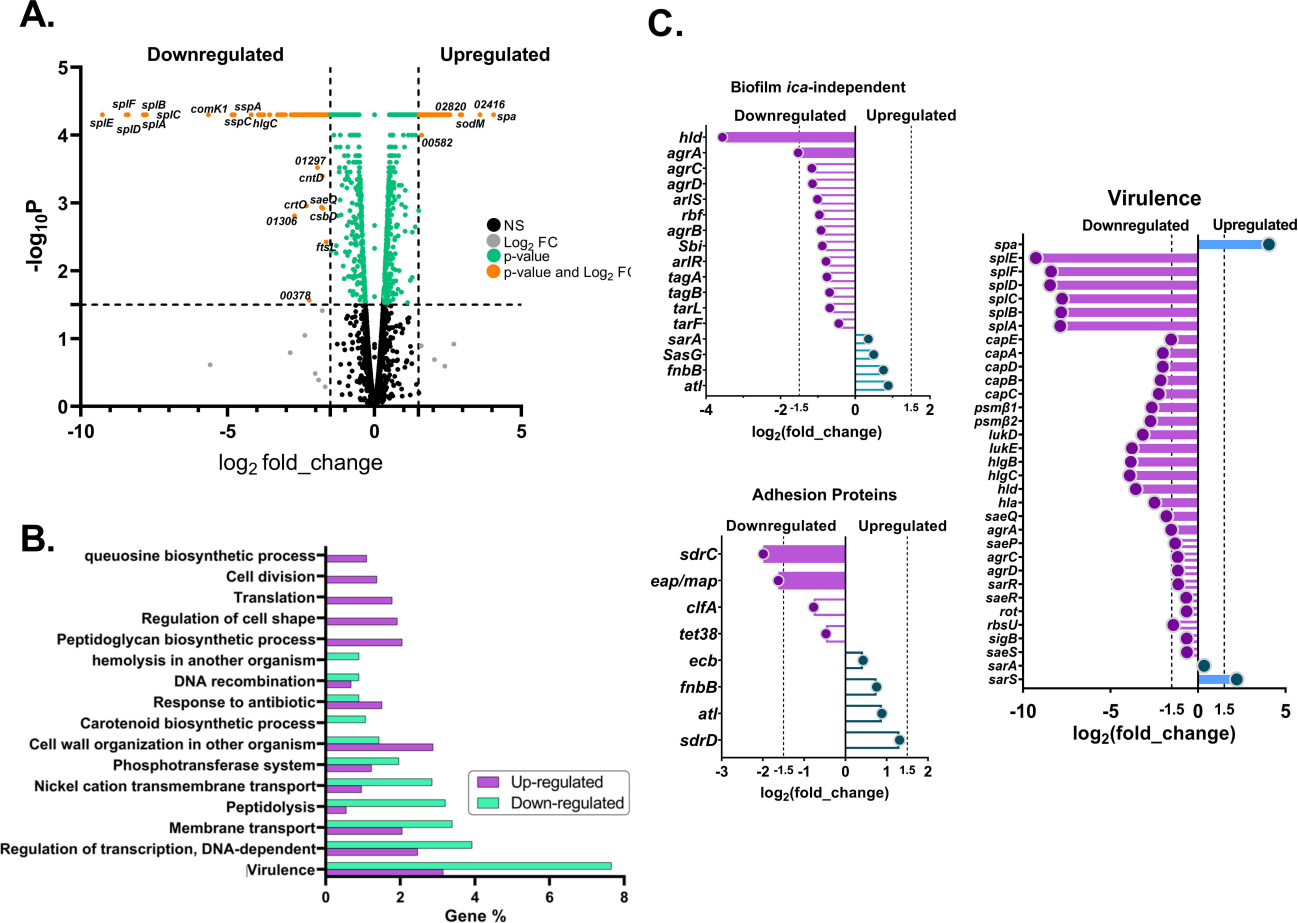
Differentially expressed genes (DEGs) in *S. aureus qrp***/***yheA*, *ymcA*, and *ylbF* mutant. (A) Volcano plot of the genes detected in the exponential phase. Each dot represents a gene, with orange dots highlighting differentially expressed genes (DEGs). The right and left sides indicate upregulated and downregulated genes, respectively. (B) Distribution of differentially expressed transcripts. The graph shows the DEGs assigned to the 17 major GO Biological Process categories. The percentage of upregulated genes is shown in blue, and the percentage of downregulated genes is shown in purple. (C) Differentially expressed genes involved in biofilm formation via* ica*-independent pathway, adhesion proteins, and virulence. The dotted lines indicate the detection limit for transcripts with genes exhibiting a log2 fold change |FC| ≥ 1.5 considered differentially expressed.

In *B. subtilis*, Com_YlbF proteins have been associated with the regulation of numerous genes by influencing the activation of master transcription factors or the activity of ribonucleases (11). To further understand the function of Com_YlbF proteins in *S. aureus*, we generated a triple mutant, deletion of the *qrp*/*yheA*, *ymcA*, and *ylbF* genes, and compared the transcriptomic profile of this mutant with the wild-type strain. In this assay, we obtained data from 2,872 transcripts (with FPKMs > 0.5), covering 95% of the ORFs in the *S. aureus* genome. The results showed that 201 genes were differentially expressed (with absolute log2 fold change above the conservative threshold of 1.5) in the absence of *qrp*/*yheA*, *ymcA*, and *ylbF* (Fig. 1A). Of these, 155 genes were downregulated, and 46 were upregulated (Table S3). The top 17 biological processes associated with the differentially expressed genes in *S. aureus* are shown in Fig. 1B. Notably, the GO annotations mainly include three major categories, “virulence” (23 upregulated genes; 43 downregulated genes) [GO:0009405], “regulation of transcription DNA-dependent” (18 up; 22 down) [GO:0006355], and “membrane transport” (15 up; 19 down) [GO:0055085]. Other noble subsets include “hemolysis in another organism” (five down) [GO:0044179] and “cell adhesion” as factors involved in biofilm formation (five up) [GO:0007155] (Fig. 1A).

Notably, the transcripts for genes encoding virulence factors and biofilm formation were categorized under “virulence.” Specifically, the spl (serine-protease like) operon that harbors six serine proteases (*splE*, *splD*, *splF*, *splA*, *splB*, and *splC*) was the most downregulated (7- to 9-fold decrease), followed by hemolysins (*lukE*, *lukD*, *hlgB*, *hlgC*, *hla*, and *hld*) with a 2.4- to 3.9-fold decrease, phenol-soluble modulins (*psmβ1* and *psmβ2*) with a 2.6- to 2.7-fold decrease, fibrinogen binding protein SdrC of the MSCRAMM group (*sdrC*) with a 1.9-fold decrease, extracellular adhesion protein Eap/Map of the SERAM group (*eap/map*) with a 1.6-fold decrease, and capsular polysaccharide biosynthesis proteins (*capA*, *capB*, *capC*, *capD*, and *capE*) with a 1.5- to 2-fold decrease. Interestingly, the accessory gene regulatory Agr-system (*agrA*) also showed a decrease of 1.5-fold. On the other hand, among the upregulated and pathogenesis-categorized genes, the SarA-homologous transcriptional regulator (*sarS*) and the immunoglobulin G–binding protein A precursor (*spa*) showed the biggest change (4-fold increase) (Fig. 1A and C).

### The Qrp/YheA, YmcA, and YlbF proteins promote biofilm formation in a PIA/PNAG-dependent manner

Based on the results of the transcriptomic analysis, biofilm formation is one of the phenotypes affected by the absence of Qrp/YheA, YmcA, and YlbF proteins. To determine whether the effect on biofilm was related to any individual protein, we constructed single and double mutants in the *qrp*/*yheA*, *ymcA*, and *ylbF* genes. Deletions of these genes had no impact on growth rate (Fig. S1).

**Fig 2:**
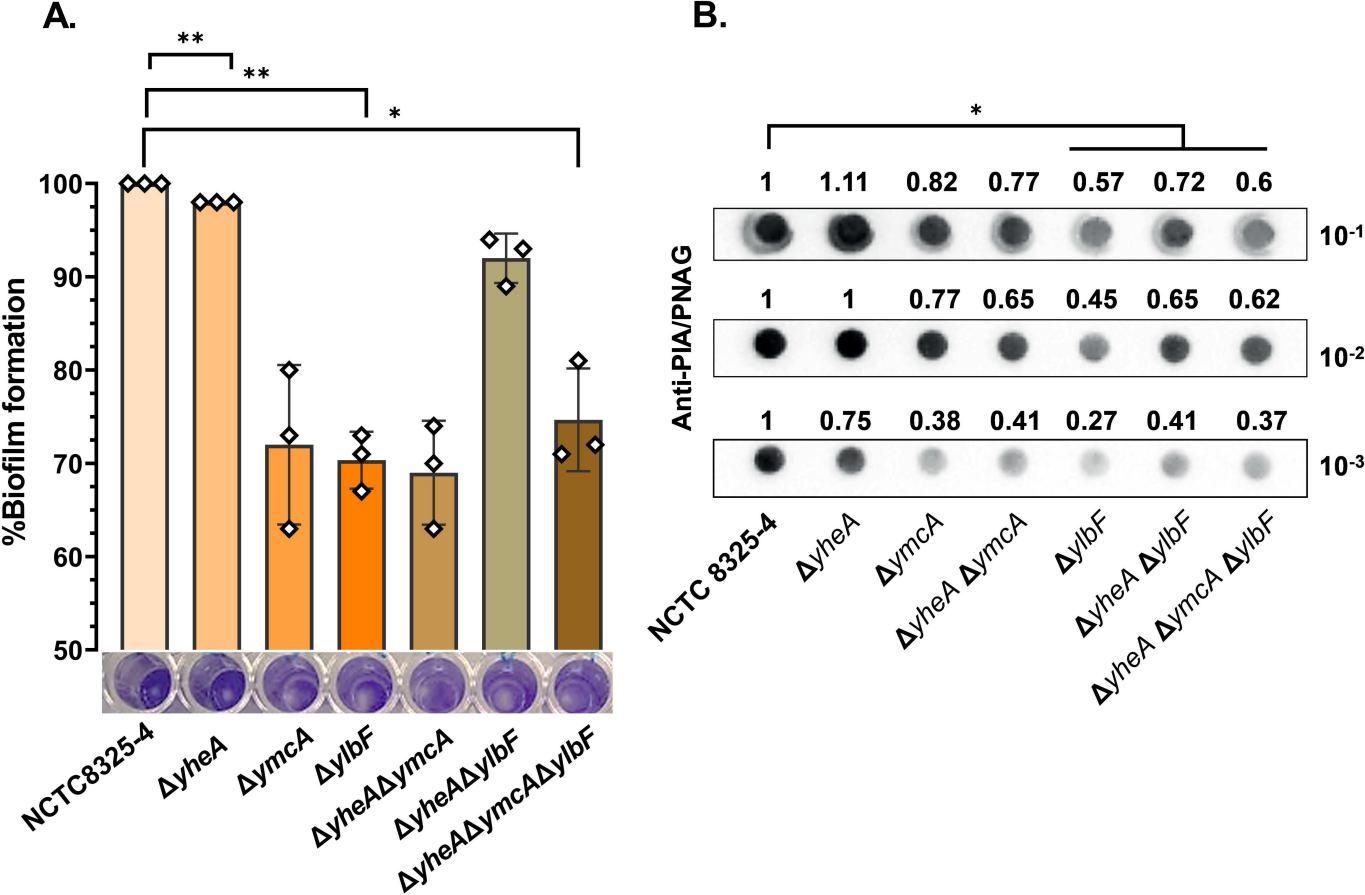
Mutants for *qrp/yheA*, *ymcA*, and *ylbF* showed a reduction of biofilm in *S. aureus*. (A) Percentage of biofilm formation relative to WT, measured as OD600 nm after crystal violet staining after 24-hour incubation in the presence of glucose. Significant reductions in biofilm formation compared to the wild-type (WT) strain were observed in Δ*ylbF* (30%, *P* value < 0.01; *P* = 0.006), Δ*ymcA* (28%, *P* value < 0.05; *P* = 0.020), Δ*qrp*/*yheA*Δ*ymcA* (31%, *P* value < 0.05; *P* = 0.037), Δ*qrp*/*yheA*Δ*ylbF* (8%, *P* value < 0.05; *P* = 0.004), and Δ*qrp*/*yheA*Δ*ymcA*Δ*ylbF* (26%, *P* value < 0.05; *P* = 0.021). (B) Dot blot densitometric analysis of PIA/PNAG in *S. aureus* NCTC8325-4 and mutants in the presence of glucose (0.25% p/v; TSBG). Significant reductions in PIA/PNAG production in Δ*ylbF* (57%, *P* value < 0.05; *P* = 0.023), Δ*qrp*/*yheA*Δ*ylbF* (40%, *P* value < 0.05; *P* = 0.048), and Δ*qrp*/*yheA*Δ*ymcA*Δ*ylbF* (47%, *P* value < 0.05; *P* = 0.026). For dot blot analysis, serial dilutions (1:10 to 1:1,000) of the total protein extraction samples were spotted onto nitrocellulose membranes, and PNAG production was detected with PIA/PNAG antibody. Relative quantification was obtained using ImageJ, and the values are expressed in densitometric proportions, normalized to wild-type (WT) strain. The assays were performed in two independent experiments, each conducted in triplicate. Δ*yheA=*Δ*qrp/yheA*. Statistical analysis was performed using a paired *t*-test. *P value < 0.05, **P value < 0.01.

We assessed the biofilm-forming ability of the mutants under high glucose concentration (0.25% p/v) and high osmolarity (3% p/v NaCl) conditions (15, 16). Most mutant strains showed reduced biofilm formation (*P* value < 0.05) under high glucose concentration, but not under high osmolarity (Fig. 2A). Significant reductions in biofilm formation compared to the wild-type (WT) strain were observed in the single mutants Δ*ylbF* (30%, *P* value < 0.01; *P* = 0.006) and Δ*ymcA* (28%, *P* value < 0.05: *P* = 0.020). The double mutants Δ*qrp*/*yheA*Δ*ymcA* (31%, *P* value < 0.05: *P* = 0.037), Δ*qrp*/*yheA*Δ*ylbF* (8%, *P* value < 0.05; *P* = 0.004), and the triple mutant Δ*qrp*/*yheA*Δ*ymcA*Δ*ylbF* (26%, *P* value < 0.05; *P* = 0.021) also showed significant reductions (Fig. 2A).

Production of polysaccharide intercellular adhesin (PIA) or polymeric *N*-acetyl-glucosamine (PNAG) by the *icaADBC* operon is tightly regulated at the transcriptional level (17). We hypothesized that the role of Com_YlbF proteins (Qrp/YheA, YmcA, and YlbF) in biofilm formation could be related to the regulation of PIA/PNAG exopolysaccharide expression. To assess changes in PIA/PNAG production, we measured the levels of this exopolysaccharide in strains cultivated in TSB supplemented with glucose (0.25% p/v; TSB_G_) using dot blot experiments with an anti-PIA/PNAG polyclonal antibody (15, 18). Our results showed significant reductions in PIA/PNAG production by 57% (*P* value < 0.05; *P* = 0.023), 40% (*P* value < 0.05; *P* = 0.048), and 47% (*P* value < 0.05; *P* = 0.026) in Δ*ylbF*, Δ*qrp/yheA*Δ*ylbF*, and Δ*qrp/yheA*Δ*ymcA*Δ*ylbF* strains, respectively (Fig. 2B). These findings suggest that YmcA and YlbF proteins are involved in the regulation of PIA/PNAG exopolysaccharide synthesis.

### The Qrp/YheA, YmcA, and YlbF proteins contribute to the hemolytic activity of *S. aureus*

**Fig 3:**
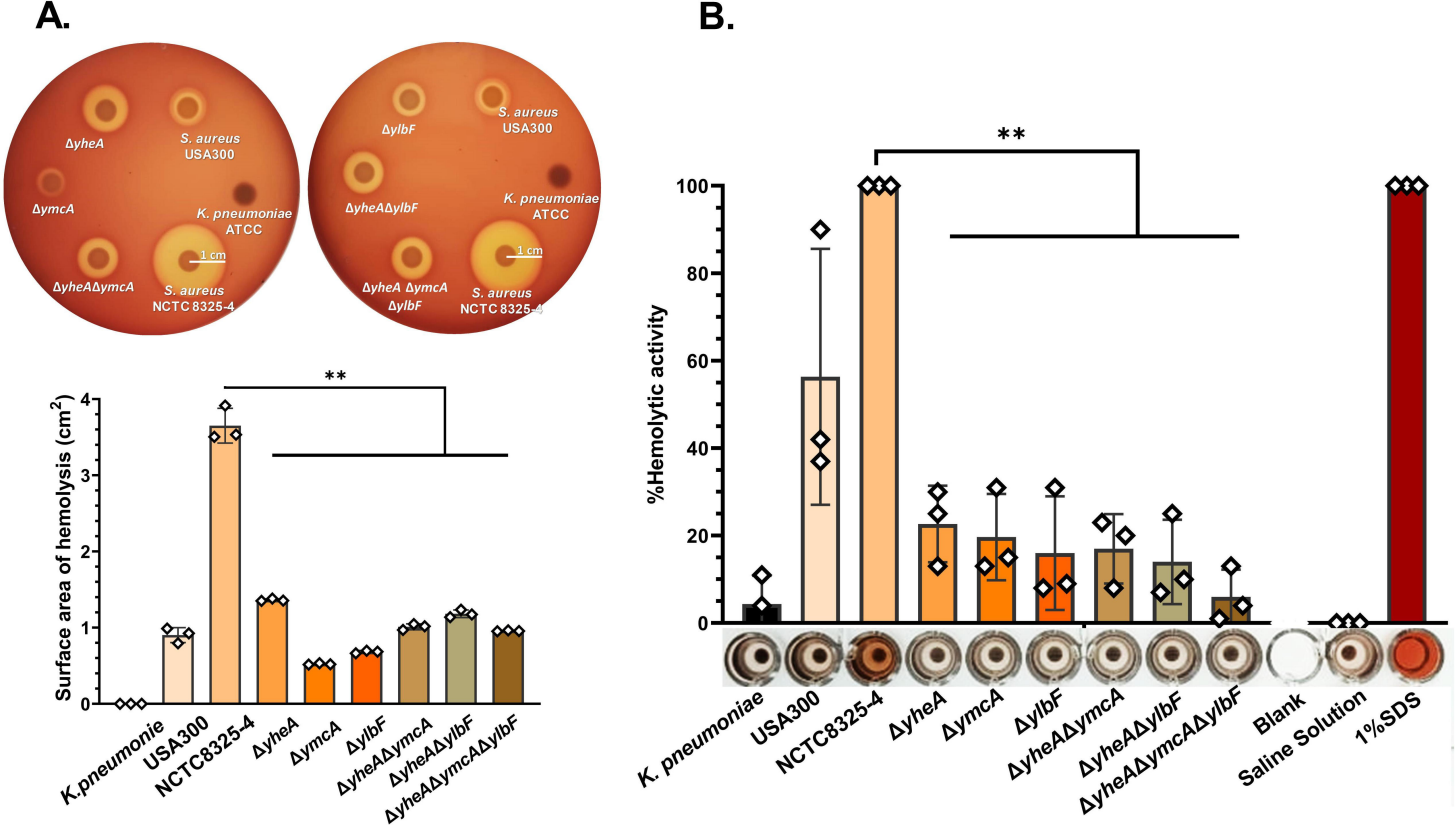
Mutants for *qrp/yheA*, *ymcA*, and *ylbF* showed a reduction of hemolysis for *S. aureus*. Assessment of the hemolytic capability by qualitative analysis (**A**) was significantly reduced in Δ*yheA* (63%, *P* value < 0.01; *P* = 0.0035), Δ*ymcA* (86%, *P* value < 0.01; *P* = 0.0019), Δ*ylbF* (81%, *P* value < 0.01; *P* = 0.0019), Δ*qrp*/*yheA*Δ*ymcA* (72%, *P* value < 0.01; *P* = 0.0033), Δ*qrp*/*yheA*Δ*ylbF* (68%, *P* value < 0.01; *P* = 0.0017), and Δ*qrp*/*yheA*Δ*ymcA*Δ*ylbF* (74%, *P* value < 0.01; *P* = 0.0024). (**B**) Quantitative analysis was significantly reduced in Δ*yheA* (77%, *P* value < 0.01;* P* = 0.0044), Δ*ymcA* (80%, *P* value < 0.01; *P* = 0.0048), Δ*ylbF* (84%, *P* value < 0.01; *P* = 0.0076), Δ*qrp*/*yheA*Δ*ymcA* (83%, *P* value < 0.01; *P* = 0.0028), Δ*qrp*/*yheA*Δ*ylbF* (86%, *P* value < 0.01; *P* = 0.0039), and Δ*qrp*/*yheA*Δ*ymcA*Δ*ylbF* (94%, *P* value < 0.01; *P* = 0.0014). The hemolytic capability of the *qrp*/*yheA*, *ymcA*, and *ylbF* mutants of *S. aureus* was drastically reduced after 24 hours of incubation. *K. pneumoniae*, non-hemolytic control. *S. aureus* USA300*,* positive control with incomplete hemolytic phenotype (SIHP). *S. aureus* NCTC8325-4*,* positive control with complete hemolytic phenotype (SCHP). Δ*yheA=*Δ*qrp/yheA*. Blank, empty well. 0.98% saline solution, negative control. 1% SDS, hemolytic positive control. Hemolytic capability quantification in blood agar plates was obtained using ImageJ. The assay was conducted once, in triplicate. The scale bar represents 1 cm. Quantification of the hemolytic capability was performed by measuring the hemoglobin released from red blood cells at 540 nm. The assay was performed in three independent experiments, each conducted in triplicate. Statistical analysis was performed using a paired *t*-test. **P value < 0.01.

The transcriptomic analysis revealed a decrease in hemolysins expression in the mutants. Red blood cells (RBCs) lysis is an important ability that *S. aureus* possesses and that has been associated with the production of different hemolysins and leukocidins, such as leukocidin ED (LukED) (19, 20). We investigated the role of *qrp/yheA*, *ymcA*, and *ylbF* mutants in the hemolytic activity of bacteria using qualitative and quantitative methods. Qualitative analysis was performed by observing opacity around individual colonies in blood agar assays (21), while quantitative analysis involved measuring the absorbance of the released hemoglobin from hemolyzed RBCs. Interestingly, in the qualitative assay, we found that the hemolytic activity of the triple-mutant strain (*qrp/yheA*, *ymcA*, and *ylbF* deletion) was significantly reduced in 74% (*P* value < 0.01; *P* = 0.0024) versus the wild-type strain NCTC8325-4. The triple-mutant strain did not produce a halo around spots of cultured cells, unlike the wild-type strain, which exhibited a transparent halo approximately 1 cm in radius (Fig. 3A). Quantitatively, hemolytic activity was reduced by 94% (*P* value < 0.01; *P* = 0.0085), with the triple-mutant strain showing minimal hemolysis of RBCs (Fig. 3B). Both results suggest that Qrp/YheA, YmcA, and YlbF proteins are involved in the regulation of hemolysins or leukocidin ED expression, probably via upregulation (20). Single-mutant strains showed an average reduction of hemolytic halo by 77% (*P* value < 0.01; *P* = 0.0076) and released hemoglobin by 80% (*P* value < 0.01; *P* = 0.0044), while double mutant strains showed an average reduction of hemolytic halo by 70% (*P* value < 0.01; *P* = 0.0074) and released hemoglobin by 85% (*P* value < 0.01; *P* = 0.0066) (Fig. 3).

### The Qrp/YheA, YmcA, and YlbF proteins contribute to the transcriptional and post-transcriptional regulation of genes through RNase-Y activity

**Fig 4:**
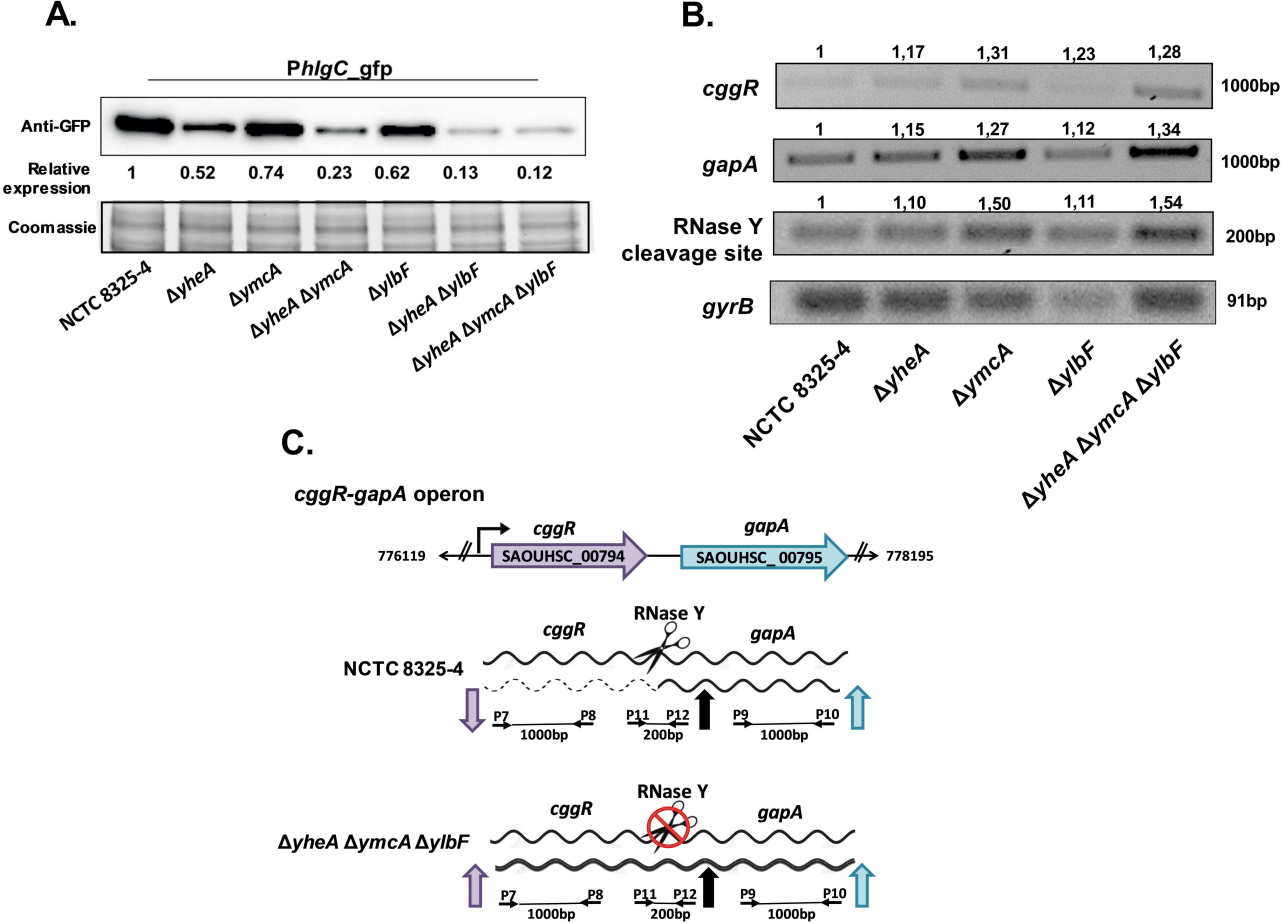
Assessment of the RNase-Y activity and *hlgC* promoter activity in *S. aureus* NCTC8325-4 and Δ*qrp***/***yheA*, Δ*ymcA*, and Δ*ylbF* strains. (A) Expression of GFP under the control of the *hlgC* promoter at stationary phase (pCN52-*hlgCp*_*gfp*) determined by Western blotting using monoclonal antibodies. Δ*yheA=*Δ*qrp/yheA*. Relative quantification of the GFP protein was obtained using the ImageJ software. The Coomassie-stained gel portion was used as a loading control. (B) Densitometric analysis of the RT-PCR of the wild-type strain NCTC8325-4 and the simple, double, and triple deletions of *qrp/yheA*, *ymcA*, and *ylbF* genes, indicating variations of the amplified regions in the *cggR-gapA* operon. The housekeeping gene *gyrB* was used for densitometric normalization. Numbers indicate the relative quantification of the PCR products obtained using the ImageJ program. The assay was performed in independent duplicates. (C) Natural scheme of bicistronic transcription regulation of the *cggR-gapA* operon via RNase-Y–mediated processing in *S. aureus* NCTC8325-4 compared to mutant strains.

According to the transcriptome results, Qrp/YheA, YmcA, and YlbF may be associated with transcriptional regulation of some virulence genes. To assess this possibility, a reporter plasmid pCN52 with a transcriptional fusion of *gfp* to the Gamma-hemolysin component C promoter (*hlgCp*) was constructed (22). The transcriptional fusion constructs were transformed into all deletion strains to monitor promoter activity by Western blotting using an anti-GFP antibody. Analysis of *hlgC* expression by promoter fusion assay using *qrp/yheA*, *ymcA*, and *ylbF* mutant strains revealed an approximately 8.3-fold reduction compared to the wild-type strain. These results suggested that the promoter-activating expression of Gamma-hemolysin (HlgC) could be modulated by Com_YlbF proteins (Fig. 4A).

In *B. subtilis*, YmcA and YlbF regulate the expression of many mRNA transcripts through the interaction with RNase-Y (10). We speculated that Com_YlbF proteins might also play a role in the post-transcriptional regulation of the virulence genes by collaborating with the activity of RNase-Y in *S. aureus*. We confirmed that RNase-Y activity was altered in the triple mutant, as this was previously observed in the *ylbF* mutant by DeLoughery et al. (11). This was evidenced by the diminished processing of the glycolytic *gapA* operon, one of the most widely studied targets of RNase-Y (23), and an increased presence of the *cggR* transcript (Fig. 4B and C).

### Com_YlbF proteins are intervening in the *S. aureus* pathogenesis *in vivo* models

**Fig 5:**
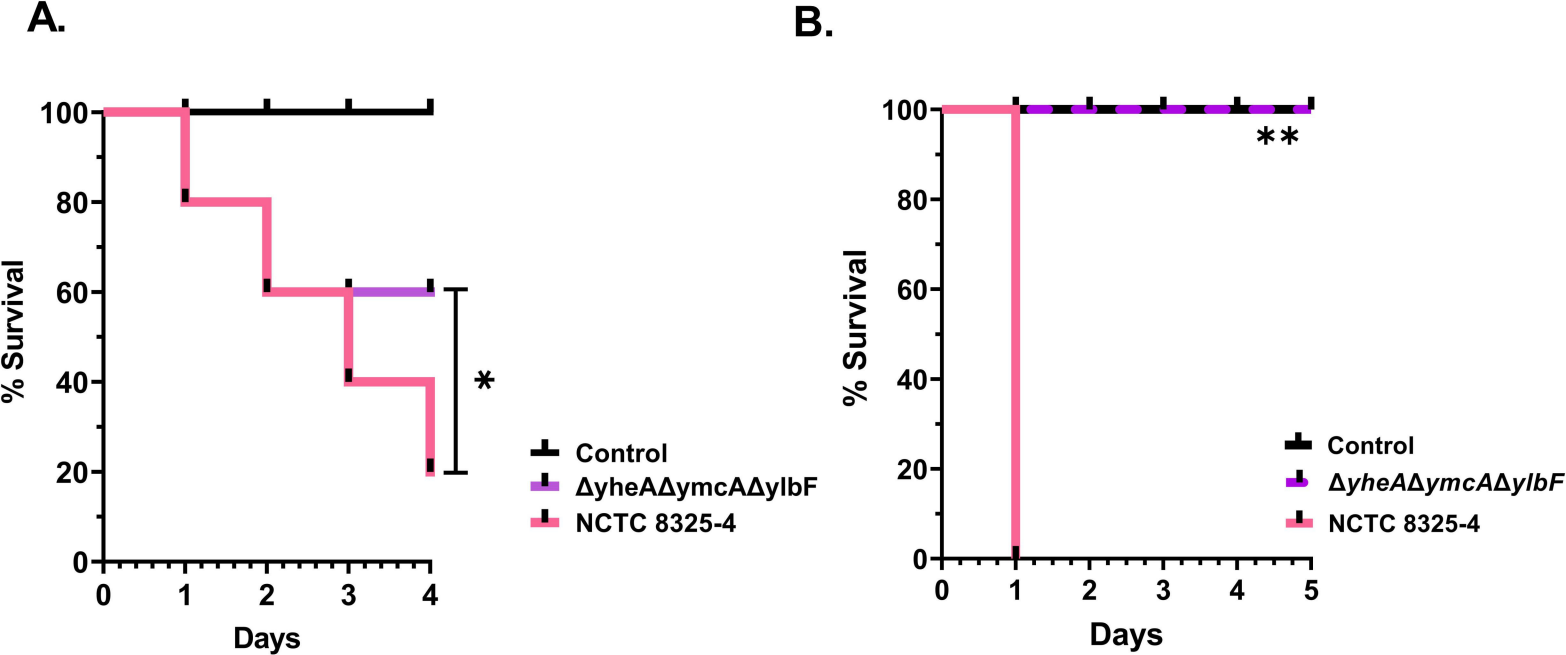
Survival analysis of *in vivo* models infected with *S. aureus*. (A) Probability of larva survival (*n* = 10 for each group) was evaluated over 4 days following inoculation. The larvae were inoculated with 5 µL of 1 × 10^5^ CFU of the *S. aureus* NCTC8325-4 and *qrp/yheA*, *ymcA,* and *ylbF* deletion strain (OD600 at 0.1). Statistical analysis was performed using the log-rank test. *P value < 0.05. This assay was performed in three independent experimental replicates. (B) The probability of mouse survival (*n* = 5 for each group) was evaluated over 5 days following intraperitoneal inoculation with 150 µL of 5 × 10^8^ CFU of the *S. aureus* NCTC8325-4 and *qrp/yheA*, *ymcA*, and *ylbF* deletion strain (OD600 at 0.7). Statistical analysis was performed by log-rank (Mantel-Cox) test. **P value < 0.01. This assay was performed once. Δ*yheA=*Δ*qrp/yheA*. The inoculation with saline solution (0.98%) was used as a control.

In order to assess whether the phenotypic alterations found *in vitro* could affect the *S. aureus* pathogenesis, we performed a *Galleria mellonella* infection model and a murine peritonitis model. For the larval model, they were exposed to different inoculum sizes of both the wild-type *S. aureus* strain and the corresponding isogenic mutants for *qrp/yheA*, *ymcA*, and *ylbF*. Mortality rates were monitored over 4 days. The larvae were injected with 5 µL containing 1 × 10^5^ CFU, allowing us to calculate a 50% mortality rate by the fourth day and enabling us to observe larval health indicators during the study. Survival rates in *G. mellonella* with 5 × 10^5^ CFU revealed a 40% mortality rate among larvae exposed to the mutant strains compared to 80% mortality with the wild-type strain (Fig. 5A). The changes in survival rates were visibly different between the wild-type and triple-mutant strains and significantly reduced in 40% (*P* value < 0.05; *P* = 0.0397).

The mice were injected intraperitoneally (i.p.) with 150 µL containing 5 × 10^8^ CFU of the mutant strain Δ*qrp*/*yheA*Δ*ymcA*Δ*ylbF *and the wild-type NCTC8325-4 strain (24–26)*.* The mice inoculated with the mutant strain Δ*qrp*/*yheA*Δ*ymcA*Δ*ylbF* showed similar results to the negative control, with 100% survival over the 5-day observation period. In contrast, injection of the wild-type strain NCTC8325-4 resulted in all infected mice developing a lethal infection within 24 hours (Fig. 5B). The changes in survival rates were visibly different between the wild-type and triple-deletion strains and significantly reduced in 100% (*P* value < 0.01; *P* = 0.0009). Both *in vivo* models showed that the deletion of Com_YlbF (Qrp/YheA, YmcA, and YlbF) proteins in *S. aureus* significantly increases the survival rate of larvae and mice.

## DISCUSSION

In the present study, we reveal some roles that Qrp/YheA, YmcA, and YlbF proteins may have in *S. aureus*. They seem to be participating in an RNase-Y–involved way, necessary to stimulate the adequate production of virulence factors, such as hemolysins, proteases, adhesion proteins, and PIA/PNAG. Most of the studies have been focused on deciphering the role of the proteins YmcA and YlbF in *B. subtilis*, showing that these are necessary for the K-state, sporulation, and normal biofilm formation (7, 8, 10, 11, 27). However, in *S. aureus*, the knowledge on these Com_YlbF proteins is scarce. Only it has been reported that the full *ylbF* gene deletion affects the enzymatic activity of the endoribonuclease RNase-Y (11).

Additionally, we examined in both *G. mellonella* and murine peritonitis models the effect of Qrp/YheA, YmcA, and YlbF proteins in *S. aureus* and the possible relationship of the expression of *S. aureus* virulence factors during infection. Notably, the triple-mutant strain had also attenuated virulence in both *G. mellonella* and mouse infection assays, perhaps due to lack of bacterium ability to generate some virulence factors. To highlight, similar results were reported by Kaito et al. (28): when the *rny*/*cvfA* gene (encoding RNase-Y protein) was disrupted in the CK501 strain of *S. aureus*, a decreased secretion of exotoxins, reduced expression of RNAII, RNAIII, and other virulence genes, and virulence attenuation in silkworm infection model were observed (28). Several studies have shown the important role that diverse virulence factors have during the course of Staphylococcal infection and their ability to cause disease in human patients and animal model infections (29). The survival results suggest that the mRNA levels regulated by RNase-Y in the mutant strain could be related to low mortality in larvae or lack of mortality in mice. Although *G. mellonella* lacks an adaptive immune system, it has been an informative model for pathogen-host interactions, as its innate system (cellular and humoral response) involves hemocyte immune cells, which resemble mammalian neutrophils (30, 31). We hypothesize that Com_YlbF proteins could be involved in systemic involvement in an infectious model of *G. mellonella*. This is supported by the increased survival time observed in larvae infected with the mutant strain of *S. aureus*, compared with the WT. Possibly, the decreased expression of pathogen virulence factors such as serine proteases and capsular polysaccharide proteins could stimulate the cellular and humoral response.

In addition, the transcriptomic results showed that the most relevant number of altered transcripts was in the category “virulence” (5% decreased; 2% increased). Our transcriptional analysis also identified a large decrease in the *spl* operon in the triple mutant (between 7- and 9-fold). Interestingly, the expression of serine protease encoding in this operon (that has only been found in *S. aureus*) has been associated with affectations of the host immune responses. For instance, Paharik et al. (32), examining the behavior of a *spl* mutant in rabbit pneumonia model, revealed a complex virulence phenotype, where the Spl absence did not produce attenuated lethality but was able to induce lung-specific severe damage. In addition, they also found that the SplA was able to cleave human mucin 16 (first host substrate identified for a Spl protein), an important protective protein of the epithelia (32). This evidence suggests the role of the *spl* operon as a virulence factor in *S. aureus* disease and might correlate to the no mortality mice found by us.

Other groups of genes playing an important role in the virulence, resistance, and survival of *S. aureus* are genes encoding capsular polysaccharide biosynthesis proteins (CP), major factors in bacterial evasion of the host immune defenses (33, 34). The *S. aureus* CPs have been shown to possess antiphagocytic properties, allowing the bacterium to persist in the blood and tissues of infected hosts (35). Iqbal et al. (36) described the transcriptome of a clinical strain derived from animals, compared it with a human *S. aureus* virulent strain, and reported a significant upregulation in *cap* genes, highlighting their pathogenicity in a murine model (36). Similarly, our transcriptional analyses of the Com_YlbF mutant showed that the cap operon expression levels were significantly decreased (between 1.5- and 2-fold), suggesting favorable conditions for phagocytosis. This may enhance the cellular defense response by facilitating the internalization and destruction of the pathogen by hemocyte immune cells.

*Staphylococcus aureus* strains encode between 16 and 17 different two-component systems (37–39). Some of them were affected in our transcriptional analyses, such as *lytRS* (murein hydrolase activity), *vraSR* (cell wall biosynthesis), *saeRS* (secreted factors mainly involved in immune evasion), and *arlRS*, while *kdpED* (potassium transport) and *agrAC* (cell wall protein synthesis and detection of quorum sensing) were deregulated. The best-studied of these regulatory systems is the accessory gene regulator *agr,* which acts as a master virulence regulator (40) and plays a key role in biofilm formation in both *S. aureus* and *S. epidermidis* (41, 42). Although numerous biofilm-related genes were repressed, many genes did not pass the threshold, but we identified the greatest transcriptional impact associated with this master regulator and the SarA protein family of transcriptional regulators (SarARXZ, ArlRS, CcpA, CodY, Rot, and SrrAB), as well as the alternative sigma factor (SigB) (Table S3) (43–46).

The Com_YlbF proteins (YmcA and YlbF) gained attention for their essential role in biofilm formation in *B. subtilis* (9). Then, our first focus was to assess whether this same impact could be seen in *S. aureus,* addressing both biofilm synthesis mechanisms that have been proposed: the *ica*-dependent pathway, which is based on a PIA/PNAG production and activated by under osmotic stress conditions (NaCl) (5, 16, 47, 48), and the *ica*-independent pathway that apparently involves adhesion proteins and is activated by under conditions of acidity (glucose) (49). Here, we have found that single-, double-, and triple-mutant strains do not abolish biofilm ability but only reduce it in the ica-independent pathway.

One of the main attributes of *S. aureus* is the ability to evade both innate and adaptive immune responses involving several virulence factors, including exotoxins (50). *In vitro* and *in vivo* animal studies have shown that pore-forming toxins (PFTs) are the main virulence factors involved in the pathophysiology of Staphylococcal infections. However, their regulatory mechanisms still need to be further explored. To confirm the hypothesis of Com_YlbF protein domains and their participation in virulence, we evaluated the triple-mutant strain in a qualitative and quantitative hemolytic assay using sheep red blood cells, demonstrating their high participation in virulence hemolytic capacity of *S. aureus*. Our data suggest that the Qrp/YheA, YmcA, and YlbF proteins act by modulating virulence and pathogenicity in *S. aureus*. Our results show a decrease in the production of several virulence factors but also a smaller decrease in biofilm formation in the triple-mutant strain. This phenomenon could be explained for a potential destabilization in the biofilm integrity due to lower availability of virulence factor into the extracellular matrix of the bacterium. This model was proposed by Graf et al. (51), who discovered how various secreted virulence factors, such as hemolysins, leukotoxins, lipases, and ribosomal proteins, could be key components for *S. aureus* biofilm integrity (51).

In the gene expression processes, ribonucleases (RNases) are proteins responsible for post-transcriptional regulation of RNA maturation (52, 53), with RNase-Y being the main ribonuclease implied in the degradation or stability (in several cases) of RNA transcripts (14, 53, 54). Regarding *S. aureus*, an ortholog of RNase-Y (*rny*/*cvfA*) was discovered by Kaito et al. (28, 55), but, to date, its characterization is very limited. A study performed by Khemici et al. (13) showed that at least 99 genes could be processed for this endoribonuclease, including mRNA and small non-coding RNAs. We carried out a comparative analysis of the transcripts reported by Khemici et al. (13). The transcriptional analysis of the study strain, *S. aureus Δqrp/yheAΔymcAΔylbF*, is similar to that of Khemici et al. (13) for *S. aureus* N315, where some ORFs associated with the RNase-Y cleavage site were also stabilized in the mutant strain (Table S4).

The enzyme participates more frequently in the degradation of many RNAs but also in the stabilization of others (54, 56), and their deletion causes a decrease in the virulence of the bacteria *in vivo* models (decreased hemolysis, adhesion, and activation of some metabolic pathways) (22, 57, 58). To highlight, some studies carried out by the Wolz lab have found that RNase-Y is important for the stabilization of the immature transcript of the operon SaePQRS and other virulence-related small RNAs (22, 57, 59). The operon SaePQRS is a two-component system that acts as a global regulator of the expression of major virulence genes in *S. aureus*, including hemotoxins (60–62).

In *B. subtilis*, an example of a multigene transcript that undergoes endoribonuclease-dependent maturation is the *cggR-gapA* glycolysis operon (11). With the Rend-seq assay in *S. aureus*, DeLoughery et al. (11) demonstrated that the generation of one of the three mRNA isoforms of the *cggR*(*gapR*)-*gapA* operon requires the YlbF ortholog (11, 63–65). Recently, Le Scornet et al. (66) identified critical factors for precise and efficient RNA cleavage by RNase-Y (for instance, a secondary structure with a few nucleotides downstream of the RNase y-cleavage site) (66). Our results in *S. aureus* indicated that in the triple-deletion strain, the transcript of the *cggR-gapA* operon is not adequately processed, suggesting a lower activity of RNase-Y ribonuclease. As previously mentioned, this change in the regulation of the *cggR-gapA* operon in *S. aureus* is likely a consequence of an increase in the half-life of the co-transcript, as has also been reported in *B. subtilis* (59, 67).

Taken together, it is plausible to suggest that the reduced virulence found in the triple-mutant strain could be explained by an alteration in the RNase-Y activity, which in turn affects the stabilization of operon SaePQRS and several small RNAs, producing a reduced secretion of virulence factors. The interest in exploring the role of Com_YlbF proteins has been increasing, since phylogenetic analyses have suggested that these proteins have co-evolved among Firmicutes in the Bacilli order, especially in *Bacillus subtilis* (12). In this sense, our study shows the importance of this new family of small proteins, which, although not essential for the survival of the bacterium, seem to be involved in the regulation of its biofilm formation, hemolysis, and virulence.

## MATERIALS AND METHODS

### Bacterial strains, plasmids, culture conditions, and oligonucleotides

Bacterial strains and plasmids used in this study are listed in Table S1. *Escherichia coli* strains were grown in Luria-Bertani broth (LB) (BD Difco), and *S. aureus* strains were grown in trypticase soy broth (TSB) (BD Difco). The media were supplemented, when appropriate, with 10 µL/mL erythromycin, 100 µg/mL ampicillin, 80 µg/mL 5-bromo-4-chloro-3-indolyl-β-D-galactopyranoside (X-Gal), 0.25% wt/vol glucose (TSB_G_), or 3% NaCl (TSB_N_). Oligonucleotides are listed in Table S2 and were synthesized by the Oligonucleotide Synthesis Service (Macrogen Inc., Seoul, Korea).

### Growth curves

Strain growth curves were determined by starting a pre-inoculum prepared from the strains *S. aureus* NCTC8325-4 and *qrp/yheA, ymcA*, and *ylbF* mutants in tryptic soy agar (TSA) (BD Difco). Independent colonies were taken from the cultures, and optical density (OD600) was adjusted at 0.1 absorbance units in TSA. The cultures were then incubated at 37°C with shaking at 150 rpm in TSB (BD Difco). Readings were made in a spectrophotometer GENESYS 30 (Thermo Scientific) at 600 nm for each bacterial culture, monitoring growth at intervals of approximately one hour during the first eight hours and a subsequent measurement at 33 hours. The assay was conducted once in triplicate.

### DNA manipulations and bacterial transformation

Plasmids were purified using the NucleoSpin Plasmid Miniprep Kit (Macherey-Nagel) according to the manufacturer’s protocol. FastDigest restriction enzymes and a Rapid DNA ligation kit (Thermo Scientific) were used according to the manufacturer’s instructions. Plasmids were transformed into *E. coli* IM01B strain and *S. aureus* by electroporation. Staphylococcal electrocompetent cells were generated as previously described (68, 69). Deletion mutants were generated via allelic replacement using the vector pMAD and conditions described by Arnaud et al. and Valle et al. (70, 71).

### Allelic exchange of chromosomal genes

To construct the deletions, two fragments of approximately 500 bp flanking the left and the right regions of the sequence targeted for deletion were amplified by PCR using the A, B, C, and D corresponding oligonucleotides (Table S2). Oligonucleotides to overlap have a 20-base complementary region that allows the products of the first PCR to anneal at their overlapping region, and a second PCR was performed to obtain a single fragment. The fusion products were purified and cloned into the plasmid pJET (Thermo Scientific). The fragment was then cloned into the polylinker sites of the shuttle plasmid pMAD (70) using the corresponding restriction enzymes (Table S2). The resulting pMAD (pMADr) was transformed into *E. coli* IM01B by electroporation. Plasmid pMADr contains a temperature-sensitive origin of replication and an erythromycin resistance gene. The plasmid was integrated into the chromosome through homologous recombination by growing bacteria at the non-permissive temperature (42°C) in the presence of erythromycin. From the 42°C plate, one to five colonies were picked into 10 mL of TSB and incubated for 2 to 3 days at 28°C. Tenfold serial dilutions of this culture in sterile TSB were plated on TSA containing X-Gal. White colonies, which no longer contained the pMAD plasmid, were tested to confirm the replacement by PCR using E and F corresponding oligonucleotides (Table S2) and by sequencing.

### RNA isolation, construction of cDNA libraries, and sequencing by Illumina

A bacterial pre-inoculum was prepared for the strains *S. aureus* NCTC8325-4 and *qrp/yheA*, *ymcA, *and *ylbF* mutant. They were cultivated overnight at 37°C without shaking in TSB supplemented with glucose (0.25% p/v; TSB_G_). Then, for RNA isolation, cultures were incubated with shaking until the mid-exponential phase at an OD600 of ~0.7. RNA was purified using either RNeasy Mini Kit (Qiagen) for RNA-seq and RT-PCR. The quality and quantity of the isolated RNA were determined by agarose gel electrophoresis and confirmed by measuring the absorbance at 260 nm using a Nanodrop spectrophotometer NP80 (Implen GmbH). The RNA isolation was performed on three replicates in two independent experiments. All samples were submitted to the TruSeq Total RNA Library Construction for Microbe with Ribo-Zero Kit previously to be sequenced using the NovaSeq 6000 (Illumina, CA) platform at Macrogen.

### Differentially expressed genes identification and annotation

Raw reads were generated from image data and stored in FASTQ format. Raw data were filtered to remove adaptor-contaminated and low-quality sequences and obtain clean reads. Clean reads were quality examined by the Trimmomatic program V. 0.39 (Phred score ≤20) and aligned to the reference genome of *S. aureus* NCTC8325 (NCBI Reference Sequence: NC_007795.1) using HiSat2 v. 2.1.0. Gene coverage was calculated by the percentage of genes covered by reads FPKMs (fragments per kilobase of transcript, per million mapped reads) and gene functional annotation was performed through the Cuffnorm program included in Cufflinks v2.2. The differentially expressed genes (DEGs) were identified using R DeSeq2. The genes with a *P* value < 0.05 and adjusted |log2(fold change) | >1.5 and <−1.5 were identified as DEGs, compared with the repository of the *Staphylococcus aureus* research and annotation community (AureoWiki) (72).

### Biofilm formation assay

The biofilm formation assays were performed in microtiter wells as previously described (15, 73). *S. aureus* strains were cultivated overnight in TSB supplemented with glucose (0.25% p/v; TSB_G_) to stimulate under acid conditions and NaCl (3% w/v; TSB_N_) to stimulate under osmotic stress conditions. Optical density (OD600) was adjusted at 0.1 absorbance units with 0.98% saline solution, and 5 µL was inoculated with 195 µL sterile TSB_G_ or TSB_N_ in 96-well polystyrene microtiter plates (NEST). After culturing for 24 hours at 37°C, the wells were gently washed twice with 200 mL of sterile phosphate-buffered saline (PBS). Plates were air-dried, and remaining cells adsorbed to the surface of individual wells were stained with crystal violet. The cells were quantified by solubilizing the dye with 200 µL of ethanol-acetone (80:20, vol/vol) and determining the optical density at 595 nm (OD595). Each assay was performed in triplicate and repeated at least three times.

### PIA/PNAG detection

*S. aureus* strains were cultivated overnight in TSB supplemented with glucose (0.25% p/v; TSB_G_) and incubated at 37 °C with shaking, as described by Gerke et al. (74). PIA/PNAG production was detected with an anti-*S. aureus* PIA/PNAG (D. McKenney, Boston, USA) diluted 1:10,000 (15, 18, 75) on a nitrocellulose membrane using the Bio-Dot Microfiltration kit (Bio-Rad). Bound antibodies were detected with a peroxidase-conjugated goat anti-rabbit immunoglobulin G (IgG) antibody (Jackson Immuno Research Laboratories, Inc., PA, USA) diluted 1:5,000, and Luminol Western Blotting Reagent from the Chemiluminescent Nucleic Acid Detection Kit (Thermo Scientific).

### Hemolysis assay

The hemolytic capability was evaluated using both qualitative and quantitative analyses. For the qualitative assay, overnight cultures of *S. aureus* strains were prepared by inoculating 3 mL of TSB with a single colony of each strain and incubated at 37°C without shaking. Culture absorbance was measured at OD620 and adjusted to 0.4 (McFarland 2 standard) in saline solution. A 2 µL drop of this adjusted resuspension of each *S. aureus* strain was placed in blood agar plates (5% sheep blood in tryptic soy agar [TSA], Hardy Diagnostics, CA, USA) at separation distances of 3 cm. Plates were incubated at 37°C, and the hemolytic activity was recorded at 24 hours. The presence of a distinct translucent halo around the inoculum site was considered indicative of positive hemolytic activity. The diameters of the zones of lysis and the colony were measured using a computerized image analysis system to estimate the hemolytic area.

For hemolytic capability quantitative assays, overnight cultures of *S. aureus* strains were prepared by inoculating 3 mL of TSB with a single colony of each strain independently and incubated at 37°C without shaking. Culture absorbance was measured at OD620 and adjusted to 0.4 (McFarland 2 standard) using 0.98% saline solution. The assay was performed in sheep blood. Sheep blood was defibrinated by rinsing with saline solution to obtain a 3% blood solution. A 270 µL of prepared blood was mixed with 30 µL of each previously adjusted culture of *S. aureus* strains in 96-well plates. Plates were incubated at 37°C without shaking. Hemolysis was visually manifested as the profit of the red/orange color detected by absorbance measured at 540 nm, representing the release of hemoglobin from hemolyzed RBCs. The positive control for lysis was 1% SDS, and the negative control was 0.98% saline solution. The assay was performed three times in three independent experiments.

### Analysis of post-transcriptional processing

To determine the possible involvement of Com_YlbF proteins (Qrp/YheA, YmcA, and YlbF) with processes at the post-transcriptional processes, the processing of the *cggR-gapA* operon transcript was evaluated (76, 77). RNA was purified using RNeasy Mini Kit (Qiagen), and the quality and quantity of the isolated RNA were determined as previously described. Contaminating DNA in the RNA preparations was removed using “RNase-free DNase I” (Thermo Scientific). Purified RNA samples were converted to cDNA using Moloney murine leukemia virus (M-MLV) reverse transcriptase enzyme (Promega), following the manufacturer’s instructions. cDNA (50 ng) was then used as template for RT-PCR with respective primers (Table S2). The RT-PCR products were examined by agarose gel electrophoresis and stained with ethidium bromide. The gene expression was compared between *S. aureus* NCTC8325-4 and its respective single, double, and triple *qrp/yheA*, *ymcA*, and *ylbF* mutant strains. The relative densitometry analysis was performed using ImageJ software.

### Generation of transcriptional fusions with Gfp

To obtain transcriptional fusions, the promoter of *hlgC* was amplified using the respective primers (Table S2) and cloned into pCN52 plasmid, generating pCN52-*phlgC*_*gfp* plasmids. Plasmids were transformed into *S. aureus* NCTC8325-4 and *qrp/yheA*, *ymcA*, and *ylbF* mutant strains to test for *hlgC* promoter activity. The strains were cultivated overnight at 37°C with shaking in TSB. Cells were pelleted by centrifugation at 4,500 rpm for 30 min at 4°C, washed with 1 mL of PBS, and suspended in 400 µL PBS. Then the cells were lysed using a FastPrep-24TM 5G homogenizer (MP Biomedicals, LLC, Irvine, CA, USA). Total protein extracts were recovered and analyzed by SDS-PAGE and Western blot as described by Morales-Laverde et al. (78). GFP was detected using an anti-green fluorescent protein (GFP) monoclonal antibody (Sigma) at a 1:2,500 dilution. As a secondary antibody, ECL mouse IgG conjugated with peroxidase (HRP) (Amersham) was used, diluted at 1:5,000. Antibody dilutions were performed using 0.1% PBS-Tween 5% skimmed milk solution. The Chemiluminescent Nucleic Acid Detection Kit (Thermo Scientific) was used as a substrate.

### *Galleria mellonella *survival assay

A total of 90 *Galleria mellonella* larvae were used and randomly assigned to a specific experimental group, with 30 larvae per experiment (*n* = 10 for each group). Sample size calculation was carried out using the program G*power version 3.1.9.4, based on a high effect size of 0.9, *P* value = 0.05, and a power of 0.8 (actual power was 0.8360159). The groups were evaluated in three groups: negative control (0.98% Saline Solution), positive control (wild-type strain NCTC8325-4), and mutant strain (Δ*qrp*/*yheA*Δ*ymcA*Δ*ylbF*). Healthy sixth-instar larvae, weighing between 200 and 300 mg and without specific exclusions, were obtained from the Biological Control Laboratory at the Department of Biology, Pontificia Universidad Javeriana de Bogotá (79, 80). The strains *S. aureus* NCTC8325-4 and Δ*qrp/yheA*Δ*ymcA*Δ*ylbF* mutant were prepared independently by inoculating 3 mL of TSB with a single colony of each strain independently and incubated at 37°C without shaking. The optical density of the cultures in TSB was measured at OD600 nm and adjusted with 0.98% saline solution at 0.1 (1 × 10^5^ CFU) (79, 81). Before handling the larvae, disinfection washes were carried out with hypochlorite (0.1%) and distilled sterile water. Aliquots of 5 µL of each bacterial dilution were injected into the second middle left proleg of the larvae. Control larvae were injected with the same volume (5 µL) of saline solution to monitor any problem associated with the injection process. Following injection, larvae were placed in Petri glass dishes and stored in the dark at 28°C for 4 days. For each group of larvae, their survival and appearance were evaluated in 24-hour intervals. Three independent experimental replicates were performed in triplicate. Larvae were considered dead if they did not respond to touch by moving (82). Along with the survival test, the melanization changes were recorded according to the scoring system published by Champion et al. (82).

The larva survival experiments were guided by Dr. Adriana Sáenz Aponte. The experiments were conducted in accordance with the ethical considerations of the management of invertebrate animals (83) and the protocols described in the literature (79).

### Mouse survival assay

The experiments were performed using female BALB/c mice, aged 5–6 weeks (17–22 g), with no specific exclusions, purchased from the Central Bioterium of the Universidad Nacional de Colombia. Sample size calculation was carried out using the program G*power version 3.1.9.4, based on a high effect size of 0.9, *P* value = 0.05, and a power of 0.5 (actual power was 0.5083679). The peritonitis model in BALB/c mouse strains was adapted from models previously described in the literature (24–26). A total of 15 mice (*n* = 5 for each group evaluated) were used and randomly assigned to a specific experimental group. The groups were evaluated in three groups: negative control (0.98% saline solution), positive control (wild-type strain NCTC8325-4), and mutant strain (Δ*qrp*/*yheA*Δ*ymcA*Δ*ylbF)*. For the bacterial inoculum, *S. aureus* strains were prepared by cultivating a single colony of each strain in 5 mL of TSB and incubating at 37°C without shaking. The optical density of the cultures in TSB was measured at OD600 nm and adjusted to mid-exponential phase at 0.7 with 0.98% saline solution. The mice were intraperitoneally (i.p.) injected with 150 µL of 5 × 10^8^ CFU of the *S. aureus* NCTC8325-4 and Δ*qrp*/*yheA*Δ*ymcA*Δ*ylbF* mutant strains, according to the experimental group.

The animals were housed in the animal facility at the Universidad Nacional de Colombia, in an isolated room that met all the necessary requirements for proper animal care. Groups of five female mice were kept in appropriately sized ventilated cages (500 cm² of floor space, model NexGen500, Allentown), covered with sterile wood chip (Aspen Chip and Lab Grande Aspen, NEPCO). The top of the cage held an external plastic 250 mL water bottle and a Whatman filter that allowed clean air exchange and protected the food (placed in a half pocket wire bar lid) with all the nutritional requirements for mouse survival. The cage had an enrichment (60 mm × 78 mm) for mouse entertainment. All home cages were properly labeled. During the experimental period, the animals moved to clean cages with new food and water once a day and were kept at 22°C on a 12-hour light-dark cycle.

The health status of the post-infection mice was observed for a maximum period of 5 days to minimize suffering, according to the scoring system published by Carstens et al. (84). If mice showed severe signs of illness, they were euthanized following the Guidelines for Humane Endpoints for Research, Teaching and Testing Animals (85). 

### Image quantification and statistics

GraphPad Prism software, version 9.0, was used to perform non-parametric statistical analyses showing the behavior of the variables through histograms and curves. Densitometry analysis was performed using ImageJ software (http://imagej.nih.gov/ij/). Rectangles were drawn around different bands, and the average pixel intensity was measured. The same size of the rectangle was used to quantify the bands being compared, and the density of the background of the same size was subtracted from the measurement. Two-tailed *P* values were determined based on unpaired *t*-tests or log-rank tests. The statistical significance is indicated as *P value < 0.05 and **P value < 0.01.

## Data Availability

All data generated or analyzed during this study are included in the supplemental material.

## SUPPLEMENTAL MATERIAL

The ARRIVE guidelines: Galleria mellonella jb.00061-25-s0001.pdf The ARRIVE guidelines 2.0: *Galleria mellonella* survival assay checklist.

The ARRIVE guidelines: Mice model jb.00061-25-s0002.pdf The ARRIVE guidelines 2.0: Mouse survival assay checklist.

Table S1, S2, and S4, and Figure S1 jb.00061-25-s0003.pdf Table S1: Bacterial strains and plasmids were used in this study. Table S2: Oligonucleotides used in this study. Table S3: Confirmation of the deletion of the genes. Table S4: Comparison of differentially expressed transcripts.

Table S3 jb.00061-25-s0004.xls Transcriptomic analysis of mutant strain.

ASM does not own the copyrights to Supplemental Material that may be linked to, or accessed through, an article. The authors have granted ASM a non-exclusive, world-wide license to publish the Supplemental Material files. Please contact the corresponding author directly for reuse.
