# Supplementary material for: Deciphering the function of Com_YlbF domain-containing proteins in Staphylococcus aureus
Source: J Bacteriol. 2025 Aug 18;207(9):e00061-25. doi: 10.1128/jb.00061-25 (PMC12445100; doi:10.1128/jb.00061-25)
Supplement: The ARRIVE guidelines: Galleria mellonella — The ARRIVE guidelines 2.0: Galleria mellonella survival assay checklist. [file jb.00061-25-s0001.pdf]

The ARRIVE guidelines 2.0: ***Galleria mellonella* survival assay**

Animal Research: Reporting of In Vivo Experiments

- Nathalie Percie du Sert1, Viki Hurst1, Amrita Ahluwalia2, Sabina Alam3, Marc T Avey4, Monya Baker5, William J Browne6, Alejandra Clark7, Innes C Cuthill6, Ulrich Dirnagl8, Michael Emerson9, Paul Garner10, Stephen T Holgate11, David W Howells12, Natasha A Karp13, Stanley E Lazic14, Katie Lidster1, Catriona J MacCallum15, Malcolm Macleod16, Esther J Pearl1, Ole H Petersen17, Frances Rawle18, Penny Reynolds19, Kieron Rooney20, Emily S Sena16, Shai D Silberberg21, Thomas Steckler22, Hanno Würbel23

- 1NC3Rs, UK. 2Queen Mary University of London, UK. 3Taylor & Francis Group, UK. 4ICF, USA. 5Nature, USA. 6University of Bristol, UK. 7PLOS ONE, UK. 8Charite Universitätsmedizin Berlin, Germany. 9Imperial College London, UK. 10Liverpool School of Tropical Medicine, UK. 11University of Southampton, UK. 12University of Tasmania, Australia. 13AstraZeneca, UK. 14Prioris.ai Inc, Canada 15Hindawi Ltd, UK. 16University of Edinburgh, UK. 17Cardiff University, UK. 18Medical Research Council, UK. 19University of Florida, USA. 20University of Sydney, Australia. 21National Institute of Neurological Disorders and Stroke, USA. 22Janssen Pharmaceutica NV, Belgium. 23 Universität Bern, Switzerland

The ARRIVE Essential 10

These items are the basic minimum to include in a manuscript. Without this information, readers and reviewers cannot assess the reliability of the findings.

Study design

1. For each experiment, provide brief details of study design including:

    a. The groups being compared, including control groups. If no control group has been used, the rationale should be stated.

    Response. The groups evaluated were: negative control (0.98% Saline Solution), positive control (wild-type strain NCTC8325-4) and mutant strain (Δqrp/yheAΔymcAΔylbF).

b. The experimental unit (e.g. a single animal, litter, or cage of animals).

> **Response**. 10 *G. mellonella* larvae were used for each group evaluated in the experiment.

Sample size

a. Specify the exact number of experimental units allocated to each group, and the total number in each experiment. Also indicate the total number of animals used.

> **Response**. A total of 90 *G. mellonella* larvae, 30 per experiment (n = 10 for each group).

b. Explain how the sample size was decided. Provide details of any a priori sample size calculation, if done.

Response. The program G*power versión 3.1.9.4, was used to determine the sample size needed for a paired samples t-test. The following variables were taken into account: Exposure of larvae to wild-type *S. aureus* strain can cause 100% of the animals to develop an infectious process during the time of exposure. It is likely that, in our study, the *S. aureus* triple mutant Δ*qrp*/*yheA*Δ*ymcA*Δ*ylbF* reduces the percentage of animals that develop an infectious process by 10%. Considering this assumption, we considered the two groups in independent proportions as the control group (*S. aureus* 8325-4) and the treated group (*S. aureus* mutant Δ*qrp*/*yheA*Δ*ymcA*Δ*ylbF*), assuming: the sample size needed for a paired samples t-test, an equal number of animals per group, a high effect size of 0.9, a 95% confidence level (Alpha risk) α=0.05, and the power of the analysis with a beta risk β=0.2, that is, the 80% probability that the test finds a difference. As a result, 10 individuals were estimated for the control group and 10 for the treated group to detect statistically significant differences. The number of larvae chosen for the survival assay is adequate, minimal, and necessary for the research purpose.

Inclusion and exclusion criteria

a. Describe any criteria used for including and excluding animals (or experimental units) during the experiment, and data points during the analysis. Specify if these criteria were established a priori. If no criteria were set, state this explicitly.

Response. The larvae, corresponding to the sixth instar and weighing between 200-300 milligrams. These criteria were established a priori.

b. For each experimental group, report any animals, experimental units or data points not included in the analysis and explain why. If there were no exclusions, state so.

Response. The larvae, corresponding to the sixth instar, healthy and weighing between 200-300 milligrams, with no specific exclusions, were obtained from the Biological Control Laboratory at the Department of Biology, Pontificia Universidad Javeriana de Bogotá

c. For each analysis, report the exact value of n in each experimental group.

Response. A total of 90 *G. mellonella* larvae, 30 per experiment (n = 10 each group evaluated in the experiment).

Randomisation

a. State whether randomisation was used to allocate experimental units to control and treatment groups. If done, provide the method used to generate the randomisation sequence.

> **Response**. 10 larvae were for each group used and randomly assigned to a specific experimental group. The larvae were used and randomly assigned to the specific treatment evaluated.

b. Describe the strategy used to minimise potential confounders such as the order of treatments and measurements, or animal/cage location. If confounders were not controlled, state this explicitly.

> **Response**. The groups evaluated were: negative control (0.98% Saline Solution), positive control (wild-type strain NCTC8325-4), and mutant strain (Δqrp/yheAΔymcAΔylbF). Larvae were placed in glass petri dishes and stored in the dark at 28°C for 4 days.

Blinding

Describe who was aware of the group allocation at the different stages of the experiment (during the allocation, the conduct of the experiment, the outcome assessment, and the data analysis).

> **Response**. The larvae survival experiments were advised by Dr. Adriana Sáenz Aponte, Master of Agricultural Sciences - Entomology. the experiments were conducted in accordance with the ethical considerations of the management of invertebrate animals (81) and in accordance with the protocols described in the literature (77).

References
•Crespi-Abril A-C, Rubilar T. Moving forward in the ethical consideration of invertebrates in experimentation: Beyond the Three R's Principle. Revista de Biología Tropical. 2021;69(S1):S346–S57.
•Ramarao N, Nielsen-Leroux C, Lereclus D. The insect *Galleria mellonella* as a powerful infection model to investigate bacterial pathogenesis. Journal of visualized experiments : JoVE. 2012(70):e4392.

Outcome measures

a. Clearly define all outcome measures assessed (e.g. cell death, molecular markers, or behavioural changes).

> **Response**. For each group of larvae their survival and appearance were evaluated in 24 hours intervals. Three independent experimental replicates were performed in triplicate. Larvae were considered dead if they did not respond to touch by moving if they did not

respond to touch by moving. Along with the survival test, the melanization changes were recorded according to the scoring system published by Champion et al. (2018).

References

- Champion OL, Titball RW, Bates S. Standardization of *G. mellonella* Larvae to Provide Reliable and Reproducible Results in the Study of Fungal Pathogens. J Fungi (Basel). 2018;4(3).

b. For hypothesis-testing studies, specify the primary outcome measure, i.e. the outcome measure that was used to determine the sample size.

Response. Exposure of larvae to wild-type *S. aureus* strain can cause 100% of the animals to develop an infectious process during the time of exposure. It is likely that, in our study, the *S. aureus* triple mutant Δ*qrp*/*yheA*Δ*ymcA*Δ*ylbF* reduces the percentage of animals that develop an infectious process by 10%. Considering this assumption, we considered the two groups in independent proportions as the control group (*S. aureus* 8325-4) and the treated group (*S. aureus* mutant Δ*qrp*/*yheA*Δ*ymcA*Δ*ylbF*), assuming: the sample size needed for a paired samples t-test, an equal number of animals per group, a high effect size of 0.9, a 95% confidence level (Alpha risk) α=0.05, and the power of the analysis with a beta risk β=0.2, that is, the 80% probability that the test finds a difference.

Statistical methods

a. Provide details of the statistical methods used for each analysis, including software used.

Response. GraphPad Prism software, version 9.0, was used to perform the non-parametric statistical analyses showing the behavior of the variables through histograms and curves. Densitometry analysis were performed using ImageJ software (http://imagej.nih.gov/ij/).

b. Describe any methods used to assess whether the data met the assumptions of the statistical approach, and what was done if the assumptions were not met.

Response. Two-tailed P values were determined based on unpaired t-tests or log-rank test. In images, statistical significance is indicated as *P < 0.05, **P < 0.01.

Experimental animals

a. Provide species-appropriate details of the animals used, including species, strain and substrain, sex, age or developmental stage, and, if relevant, weight.

Response. The experiments were performed using *Galleria mellonella* larvae, The larvae, corresponding to the sixth instar and weighing between 200-300 milligrams, were obtained from the Biological Control Laboratory at the Department of Biology, Pontificia Universidad Javeriana de Bogotá.

b. Provide further relevant information on the provenance of animals, health/immune status, genetic modification status, genotype, and any previous procedures.

Response. The larvae, corresponding to the sixth instar, healthy and weighing between 200-300 milligrams, were obtained from the Biological Control Laboratory at the Department of Biology, Pontificia Universidad Javeriana de Bogotá. Before handling the larvae, disinfection washes were carried out with hypochlorite (0.1%) and sterile distilled water.

Experimental procedures

For each experimental group, including controls, describe the procedures in enough detail to allow others to replicate them, including:

a. What was done, how it was done and what was used.

b. When and how often.

c. Where (including detail of any acclimatisation periods).

d. Why (provide rationale for procedures).

Response. The strains *S. aureus* NCTC8325-4 and ΔqrpyheAΔymcAΔylbF mutant, were prepared independently by inoculating 3 mL of TSB with a single colony of each strain independently and incubated at 37 °C without shaking. The optical density of the cultures in TSB, was measured at 600 nm and adjusted with 0.98% saline solution to OD 0,1 until complete the same inoculum of 1x10^5 CFU (78, 80). Before handling the larvae, disinfection washes were carried out with hypochlorite (0.1%) and sterile distilled water. Aliquots of 5 µl of each bacterial dilution were injected into the second middle left proleg of the *G. mellonella* larvae. Control larvae were injected with the same volume (5 µl) of saline solution to monitor any problem associated with the injection process. Following injection, larvae were placed in glass petri dishes and stored in the dark at 28°C for 4 days. For each group of larvae their survival and appearance were evaluated in 24 hours intervals. Three independent experimental

replicates were performed in triplicate. Larvae were considered dead if they did not respond to touch by moving if they did not respond to touch by moving. Along with the survival test, the melanization changes were recorded according to the scoring system published by Champion et al. (2018).

References

- Champion OL, Titball RW, Bates S. Standardization of *G. mellonella* Larvae to Provide Reliable and Reproducible Results in the Study of Fungal Pathogens. J Fungi (Basel). 2018;4(3).

Results

For each experiment conducted, including independent replications, report:

a. Summary/descriptive statistics for each experimental group, with a measure of variability where applicable (e.g. mean and SD, or median and range).

Response. Probability of larvae survival (n = 10 for each group) was evaluated over 4 days following inoculation. Statistical analysis was performed using log-rank test *P < 0.05. This assay was performed in three independent experimental replicates.

b. If applicable, the effect size with a confidence interval.

Response. not applicable
