# Supplementary material for: Deciphering the function of Com_YlbF domain-containing proteins in Staphylococcus aureus
Source: J Bacteriol. 2025 Aug 18;207(9):e00061-25. doi: 10.1128/jb.00061-25 (PMC12445100; doi:10.1128/jb.00061-25)
Supplement: The ARRIVE guidelines: Mice model — The ARRIVE guidelines 2.0: Mouse survival assay checklist. [file jb.00061-25-s0002.pdf]

The ARRIVE guidelines 2.0: **Mice survival assay**

Animal Research: Reporting of In Vivo Experiments

- Nathalie Percie du Sert1, Viki Hurst1, Amrita Ahluwalia2, Sabina Alam3, Marc T Avey4, Monya Baker5, William J Browne6, Alejandra Clark7, Innes C Cuthill6, Ulrich Dirnagl8, Michael Emerson9, Paul Garner10, Stephen T Holgate11, David W Howells12, Natasha A Karp13, Stanley E Lazic14, Katie Lidster1, Catriona J MacCallum15, Malcolm Macleod16, Esther J Pearl1, Ole H Petersen17, Frances Rawle18, Penny Reynolds19, Kieron Rooney20, Emily S Sena16, Shai D Silberberg21, Thomas Steckler22, Hanno Würbel23

- 1NC3Rs, UK. 2Queen Mary University of London, UK. 3Taylor & Francis Group, UK. 4ICF, USA. 5Nature, USA. 6University of Bristol, UK. 7PLOS ONE, UK. 8Charite Universitätsmedizin Berlin, Germany. 9Imperial College London, UK. 10Liverpool School of Tropical Medicine, UK. 11University of Southampton, UK. 12University of Tasmania, Australia. 13AstraZeneca, UK. 14Prioris.ai Inc, Canada 15Hindawi Ltd, UK. 16University of Edinburgh, UK. 17Cardiff University, UK. 18Medical Research Council, UK. 19University of Florida, USA. 20University of Sydney, Australia. 21National Institute of Neurological Disorders and Stroke, USA. 22Janssen Pharmaceutica NV, Belgium. 23 Universität Bern, Switzerland

The ARRIVE Essential 10

These items are the basic minimum to include in a manuscript. Without this information, readers and reviewers cannot assess the reliability of the findings.

Study design

1. For each experiment, provide brief details of study design including:

    a. The groups being compared, including control groups. If no control group has been used, the rationale should be stated.

    Response. The groups evaluated were: negative control (0.98% Saline Solution), positive control (wild-type strain NCTC8325-4) and mutant strain (Δqrp/yheAΔymcAΔylbF).

b. The experimental unit (e.g. a single animal, litter, or cage of animals).

Response. Groups of five female BALB/c mice were used for each treatment in the experiment.

Sample size

a. Specify the exact number of experimental units allocated to each group, and the total number in each experiment. Also indicate the total number of animals used.

Response. A total of 15 mice, (n = 5 for each group evaluated), were used and randomly assigned to a specific experimental group.

b. Explain how the sample size was decided. Provide details of any a priori sample size calculation, if done.

Response. The program G*power versión 3.1.9.4, was used to determine the sample size needed for a paired samples t-test. The following variables were taken into account: Exposure of mice to wild-type *S. aureus* strain can cause 100% of the animals to develop an infectious process during the time of exposure. It is likely that, in our study, the *S. aureus* triple mutant Δ*qrp*/*yheA*Δ*ymcA*Δ*ylbF* reduces the percentage of animals that develop an infectious process by 10%. Considering this assumption, we considered the two groups in independent proportions as the control group (*S. aureus* 8325-4) and the treated group (*S. aureus* mutant Δ*qrp*/*yheA*Δ*ymcA*Δ*ylbF*), assuming: the sample size needed for a paired samples t-test, an equal number of animals per group, a high effect size of 0.9, a 95% confidence level (Alpha risk) α=0.05, and the power of the analysis with a beta risk β=0.5, that is, the 50% probability that the test finds a difference. As a result, 5 individuals were estimated for the control group and 5 for the treated group to detect statistically significant differences. The number of mice chosen for the survival assay is adequate, minimal, and necessary for the research purpose.

Inclusion and exclusion criteria

a. Describe any criteria used for including and excluding animals (or experimental units) during the experiment, and data points during the analysis. Specify if these criteria were established a priori. If no criteria were set, state this explicitly.

Response. Experiments were performed using female BALB/c mice, aged 5–6 weeks (17–22 g). these criteria were established a priori.

b. For each experimental group, report any animals, experimental units or data points not included in the analysis and explain why. If there were no exclusions, state so.

Response. The experiments were performed using female BALB/c mice, aged 5–6 weeks (17–22 g), with no specific exclusions, purchased from the Central Bioterium of the Universidad Nacional de Colombia

c. For each analysis, report the exact value of n in each experimental group.

Response. A total of 15 female BALB/c mice, n = 5 for each treatment evaluated in the experiment.

Randomisation

a. State whether randomisation was used to allocate experimental units to control and treatment groups. If done, provide the method used to generate the randomisation sequence.

Response. A total of 15 mice, n = 5 for each group evaluated and were used and randomly assigned to the specific treatment evaluated.

b. Describe the strategy used to minimise potential confounders such as the order of treatments and measurements, or animal/cage location. If confounders were not controlled, state this explicitly.

Response. The groups evaluated were: negative control (0.98% Saline Solution), positive control (wild-type strain NCTC8325-4) and mutant strain (Δqrp/yheAΔymcAΔylbF). The animals were housed in the animal facility at Universidad Nacional de Colombia, in an isolated room meeting all the necessary requirements for proper animal care. Groups of five female mice were kept in appropriately sized ventilated cages (500 cm^2 of floor space, model NexGen500, Allentown), covered with sterile wood chip (Aspen Chip and Lab Grande Aspen, NEPCO). The top of the cage held an external plastic 250 mL water bottle and a Whatman filter that allowed clean air exchange and protected the food (placed in a half pocket wire bar lid) with all the nutritional requirements for mice's survival . The cage had an enrichment (60 mm x 78 mm) for mice entertainment. All home cages were properly labeled. During the experimental period, the animals moved to clean cages with new food and water once a day and were kept in the experimental 22°C, on a 12-h light-dark cycle.

Blinding

Describe who was aware of the group allocation at the different stages of the experiment (during the allocation, the conduct of the experiment, the outcome assessment, and the data analysis).

Response. The mouse survival experiments were directed by Dr. Myriam L. Velandia-Romero, author, and in charge of the animals throughout the experimental process. who is certified by the Colombian Association for the Science and Welfare of Laboratory Animals (ACCBAL), the Institutional Committees for the Care and Use of Animals (CICUA) of the Pontificia Universidad Javeriana and the Universidad de los Andes, and the Research Ethics Committee of the National University of Colombia.

Outcome measures

a. Clearly define all outcome measures assessed (e.g. cell death, molecular markers, or behavioural changes).

> **Response**. The health status of the postinfection mice was observed for a maximum period of 5 days to minimize suffering, according to the scoring system published by Carstens, *et al.* (2000). If mice showed severe signs of illness, they were euthanized following Guidelines-Humane Endpoints for Research, Teaching and Testing Animals (2002).
>
> References
>
> •Carstens E, Moberg GP. Recognizing pain and distress in laboratory animals. ILAR J. 2000;41(2):62-71.
> •Stokes WS. Humane Endpoints for Laboratory Animals Used in Regulatory Testing. ILAR Journal. 2002;43(Suppl_1): S31-S8.

b. For hypothesis-testing studies, specify the primary outcome measure, i.e. the outcome measure that was used to determine the sample size.

> **Response**. Exposure of mice to wild-type *S. aureus* strain can cause 100% of the animals to develop an infectious process during the time of exposure. It is likely that, in our study, the *S. aureus* triple mutant Δ*qrp*/*yheA*Δ*ymcA*Δ*ylbF* reduces the percentage of animals that develop an infectious process by 10%. Considering this assumption, we considered the two groups in independent proportions as the control group (*S. aureus* 8325-4) and the treated group (*S. aureus* mutant Δ*qrp*/*yheA*Δ*ymcA*Δ*ylbF*), assuming: the sample size needed for a paired samples t-test, an equal number of animals per group, a high effect size of 0.9, a 95% confidence level (Alpha risk) α=0.05, and the power of the analysis with a beta risk β=0.5, that is, the 50% probability that the test finds a difference.

Statistical methods

a. Provide details of the statistical methods used for each analysis, including software used.

> **Response**. GraphPad Prism software, version 9.0, was used to perform the non-parametric statistical analyses showing the behavior of the variables through histograms and curves. Densitometry analysis were performed using ImageJ software (http://imagej.nih.gov/ij/).

b. Describe any methods used to assess whether the data met the assumptions of the statistical approach, and what was done if the assumptions were not met.

Response. Two-tailed P values were determined based on unpaired t-tests or log-rank test. In images, statistical significance is indicated as *P < 0.05, **P < 0.01.

Experimental animals

a. Provide species-appropriate details of the animals used, including species, strain and substrain, sex, age or developmental stage, and, if relevant, weight.

Response. The experiments were performed using female BALB/c mice, aged 5–6 weeks (17–22 g), with no specific exclusions, purchased from the Central Bioterium of the Universidad Nacional de Colombia.

b. Provide further relevant information on the provenance of animals, health/immune status, genetic modification status, genotype, and any previous procedures.

Response. The experiments were performed using female BALB/c mice, aged 5–6 weeks (17–22 g), with no specific exclusions, purchased from the Central Bioterium of the Universidad Nacional de Colombia.

Experimental procedures

For each experimental group, including controls, describe the procedures in enough detail to allow others to replicate them, including:

a. What was done, how it was done and what was used.

b. When and how often.

c. Where (including detail of any acclimatisation periods).

d. Why (provide rationale for procedures).

Response. The peritonitis model in BALB/c mouse strains was adapted from models previously described in the literature (Rauch S, *et al.* 2012; Domenech A, *et al.* 2004; Frimodt-Moller N.1993). A total of 15 mice, (n = 5 for each group evaluated), were used and randomly assigned to a specific experimental group. The groups evaluated were: negative control (0.98% Saline Solution), positive control (wild-type strain NCTC8325-4) and mutant strain (Δqrp/yheAΔymcAΔylbF). For the bacterial inoculum, S. aureus strains were prepared by cultivating a single colony of each strain in 5 mL of TSB and incubating at 37 °C without

shaking. The optical density of the cultures in TSB was measured at 600 nm and adjusted to mid-exponential phase OD 0.7 with 0.98% saline solution. The mice were intraperitoneally (i.p.) injected with 150 µl 5x10^8 CFU of the S. aureus NCTC8325-4 and Δqrp/yheAΔymcAΔylbF mutant strain according to the experimental group.

The animals were housed in the animal facility at Universidad Nacional de Colombia, in an isolated room meeting all the necessary requirements for proper animal care. Groups of five female mice were kept in appropriately sized ventilated cages (500 cm^2 of floor space, model NexGen500, Allentown), covered with sterile wood chip (Aspen Chip and Lab Grande Aspen, NEPCO). The top of the cage held an external plastic 250 mL water bottle and a Whatman filter that allowed clean air exchange and protected the food (placed in a half pocket wire bar lid) with all the nutritional requirements for mice's survival . The cage had an enrichment (60 mm x 78 mm) for mice entertainment. All home cages were properly labeled. During the experimental period, the animals moved to clean cages with new food and water once a day and were kept in the experimental 22°C, on a 12-h light-dark cycle.

The health status of the postinfection mice was observed for a maximum period of 5 days to minimize suffering, according to the scoring system published by Carstens, et al. (200). If mice showed severe signs of illness, they were euthanized following Guidelines- Humane Endpoints for Research, Teaching and Testing Animals (2002).

References

•Rauch S, DeDent AC, Kim HK, Bubeck Wardenburg J, Missiakas DM, Schneewind O. Abscess formation and alpha-hemolysin induced toxicity in a mouse model of Staphylococcus aureus peritoneal infection. Infect Immun. 2012;80(10):3721-32.
•Domenech A, Ribes S, Cabellos C, Dominguez MA, Montero A, Linares J, et al. A mouse peritonitis model for the study of glycopeptide efficacy in GISA infections. Microb Drug Resist. 2004;10(4):346-53.
•Frimodt-Moller N. The mouse peritonitis model: present and future use. J Antimicrob Chemother. 1993;31 Suppl D:55-60.
•Carstens E, Moberg GP. Recognizing pain and distress in laboratory animals. ILAR J. 2000;41(2):62-71.
•Stokes WS. Humane Endpoints for Laboratory Animals Used in Regulatory Testing. ILAR Journal. 2002;43(Suppl_1): S31-S8

Results

For each experiment conducted, including independent replications, report:

a. Summary/descriptive statistics for each experimental group, with a measure of variability where applicable (e.g. mean and SD, or median and range).

Response. Probability of mice survival (n = 5 for each group) was evaluated over 5 days following intraperitoneal inoculation. Statistical analysis was performed by log-rank (Mantel-Cox) test **P < 0.01. This assay was performed once.

b. If applicable, the effect size with a confidence interval.

Response. not applicable
