# Supplementary material for: Deciphering the function of Com_YlbF domain-containing proteins in Staphylococcus aureus
Source: J Bacteriol. 2025 Aug 18;207(9):e00061-25. doi: 10.1128/jb.00061-25 (PMC12445100; doi:10.1128/jb.00061-25)
Supplement: Table S1, S2, and S4, and Figure S1 — Table S1: Bacterial strains and plasmids were used in this study. Table S2: Oligonucleotides used in this study. Table S3: Confirmation of the deletion of the genes. Table S4: Comparison of differentially expressed transcripts. [file jb.00061-25-s0003.pdf]

Tables and supplementary figures

Table S1. Bacterial strains and plasmids were used in this study.

Strains	Relevant properties	Reference
Staphylococcus aureus		
8325-4	*Staphylococcus aureus* NCTC 8325-4 prophage curing and harbors an 11 bp deletion in the *rsbU* gene	(1)
∆*yheA*	*S. aureus* 8325-4 with *qrp/yheA gene deleted*	This study.
∆*yheA* ∆*ylbF*	*S. aureus* 8325-4 with *qrp/yheA,* and *ylbF genes deleted*	This study
∆*yheA* ∆*ymcA*	*S. aureus* 8325-4 with *qrp/yheA. and ymcA genes deleted*	This study
∆*yheA* ∆*ymcA* ∆*ylbF*	*S. aureus* 8325-4 with *qrp/yheA, ymcA,* and *ylbF genes deleted*	This study
8325-4 pCN52-Pgfp	*S. aure*us 8325-4, with pCN52 and expression of GFP	This study
8325-4 pCN52-PhlgC	*S. aure*us 8325-4, with pCN52 plasmid with the gamma hemolysin C subunit promoter with the GFP reporter gene.	This study
∆*yheA* ∆*ymcA* ∆*ylbF* pCN52-Pgfp	*S. aureus* 8325-4 *qrp/yheA, ymcA* y *ylbF* mutant, with pCN52 and expression of GFP under the control of the respective promoter.	This study
∆*yheA* ∆*ymcA* ∆*ylbF* pCN52-PhlgC	*S. aureus* 8325-4 *qrp/yheA, ymcA* y *ylbF* mutant, with pCN52 plasmid with the gamma hemolysin C subunit promoter with the GFP reporter gene.	This study
Escherichia coli		
IM01B	*E. coli* K12 DH10B	(2)

Plasmids	Relevant properties	Reference
pCN52	Plasmid for promoter activity coupled to the green fluorescent protein (GFP) reporter gene.	(3)
pMAD	Thermosensitive plasmid for homologous recombination in *Staphylococcus aureus*.	(4)

Table S2. Oligonucleotides used in this study.

Oligo name	Purpose	Sequence*
Plasmid for Allelic exchange of chromosomal gene		
**pMAD Fw**	Primer in the *S. aureus* ClpB promoter in pMAD	ggaagcgagaagaatcataatg
**pMAD Rv**	Primer upstream of the Multiple cloning sites (MCS) in pMAD	ctagctaatgttacgttac
Allelic exchange of chromosomal gene yheA.		
***yheA*_A Fw**	Primer forward to *SAOUHSC_01978* gene with BamHI to prove clones in pMAD.	**ggatcc**gaacttatacgtttaacatctcatcc
***yheA*_B Rv**	Primer reverse to *SAOUHSC_01978* gene.	tctatttaaaggattgaaaaatattattttaataggttatagcactcctttgttg
***yheA*_C Fw**	Primer forward with *yheB* gene and *SAOUHSC_01978* gene overlap sequence.	tattaaaataatatttttcaatcctttaaataga
***yheA*_D Rv**	Primer reverse to *yheB* gene with NcoI to prove clones in pMAD.	gg**ccatgg**tcctatgaaggctcaatcgcaccac
Allelic exchange of chromosomal gene ylbF.		
***ylbF*_A** Fw	Primer forward to *ylbC* gene with SalI to prove clones in pMAD.	gg**gtcgac**ccagaaccgtcctttaaagttgatgg
***ylbF*_B Rv**	Primer reverse to *ylbC* gene.	aaatacccctcattgttatttttctaagaaaattagtgaa
***ylbF*_C Fw**	Primer forward with *ylbC* gene overlap sequence.	caatgaggggtatttttatttattcggga
***ylbF*_D Rv**	Primer reverse to *1020* gene with NcoI to prove clones in pMAD.	gg**ccatg**gtcctatgaaggctcaatcgcaccac
***ylbF*_E Fw**	Primer forward to *ylbC* gene, for chromosomal confirmation	taatactttttaggtggttgatt
***ylbF*_F Rv**	Primer reverse to *1020* gene*,* for chromosomal confirmation	agtcagaactatgaatttgttaag
Allelic exchange of chromosomal gene ymcA.		
***ymcA*_A Fw**	Primer forward to *MiaB* gene with BamHI to prove clones in pMAD.	gg**ggatcc**ggatttagtaaaacgaatcaaagataga
***ymcA*_B Rv**	Primer reverse to *MiaB* gene*.*	tattgaatcaccatttccggc
***ymcA*_C Fw**	Primer forward to *ThiW* gene with *MiaB gene* overlap sequence.	gccggaaatggtgattcaataattacaaacaaaaaggagaacaatca
***ymcA*_D** Rv	Primer reverse to *ThiW* gene with NcoI to prove clones in pMAD.	gg**ccatg**gtgaagaataccacgtttttttagagtaa

***ymcA* _E Fw**	Primer forward to *MiaB* gene, for chromosomal confirmation	cgttataatagagatttccaaattg
***ymcA* _F Rv**	Primer reverse to *ThiW* gene, for chromosomal confirmation	aaacctaataaaatgtgtcaatag
Generation of transcriptional fusions with gfp		
***hlgC*_Fw**	Primer forward to the promoter of *hlgC* with BamHI to pCN52	cc**ggatcc**aagtaaaaattaaaagcatca
***hlgC*_Fw**	Primer reverse to the promoter of *hlgC* with KpnI to pCN52	cc**ggtacc**agaggggcaagtaagctc
**pCN_Fw**	Primer downstream of the high-copy-number ColE1/pMB1/pBR322/pUC origin of replication in pCN52	gagtgagctggcggccgctgcatg
**pCN_Rv**	Primer upstream of the gene to RepC protein in pCN52	ctgttaacttactaactctttc
**GFP_Rv**	Primer reverse to *gpf* to pCN52.	agcataggcgcgccttatttgtatagttcatccatgcca
Analysis of post-transcriptional processing		
***gapA*_Fw**	Primer forward to *gapA* gene	atggcagtaaaagtagcaattaatgg
***gapA*_Rv**	Primer reverse to *gapA* gene	cagctaagtatgctaatgtacgaac
***cggR*_Fw**	Primer forward to *cggR* gene	gtgaaagacttattgcaagcacagc
***cggR*_Rv**	Primer reverse to *cggR* gene	caagtattatctttgctgcggcttc
***cggR-gapA*_Fw**	Primer forward to amplify the cleavage region of the *cggR-gapA* operon by RNase-Y	gacgattgcacccaagaatacagtg
***cggR-gapA*_Rv**	Primer reverse to amplify the cleavage region of the *cggR-gapA* operon by RNase-Y	ctgaatgctaaacgaccaattctacc

∗The underlined nucleotides correspond to restriction enzymes.

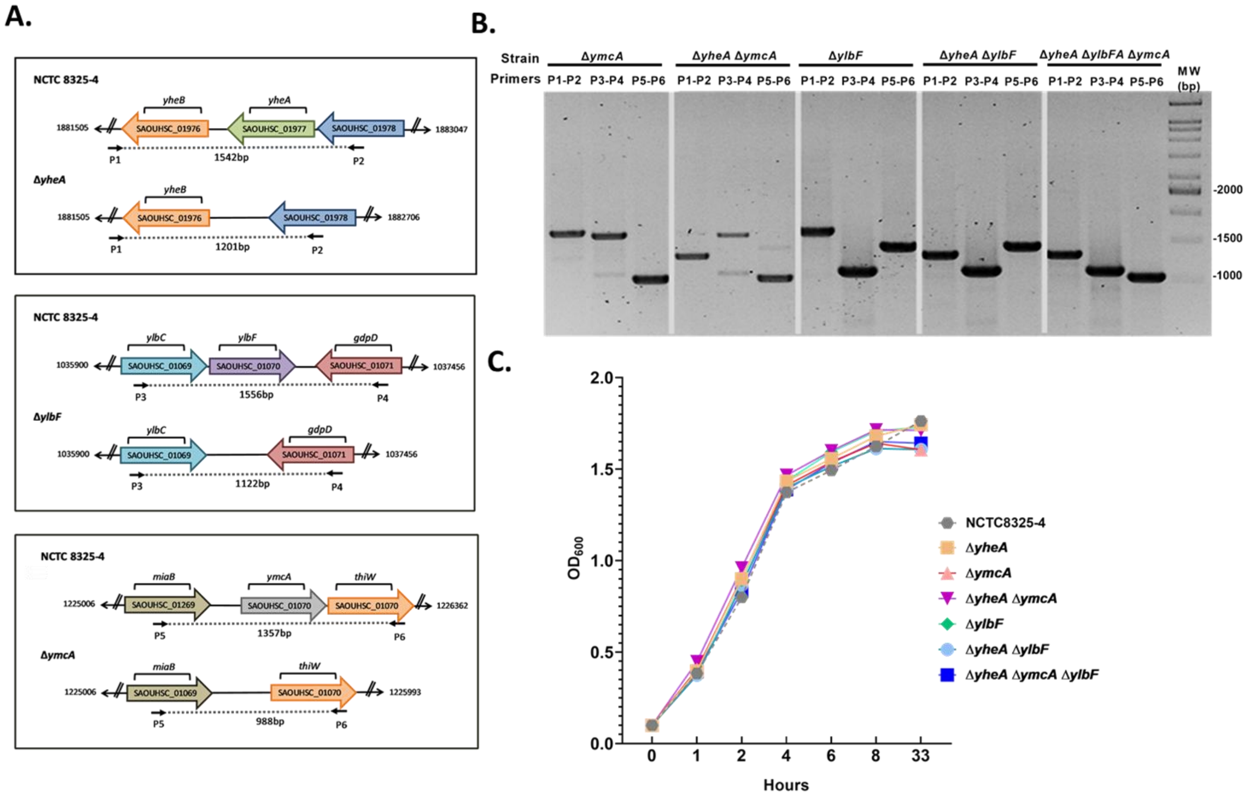

Figure S1. A. Diagram showing the positions of primers used for the identification of mutants in *S. aureus* NCTC8325-4. **B.** Confirmation of the deletion of the genes by agarose gel electrophoresis of PCR products Lane MW, 1000 bp marker. **C.** The *in vitro* growth curves of *S. aureus* NCTC8325-4 and mutation strains. The strains were cultivated in TSB overnight at 37°C. The bacterial solution was diluted to an optical density at 600 nm (OD600) of 0.1 and cultivated again. The OD600 was then measured at 1, 2, 4, 6, 8, and 33 h to draw the curves.

Table S4. Comparison of differentially expressed transcripts associated with the involvement of RNase-Y mutant (Khemici *et al.* 2015) (5) with the strain in *S. aureus* Δ*qrp/yheA*Δ*ymcA*Δ*ylbF*. The genes were compared with the repository of the *Staphylococcus aureus* research & annotation community (AureoWiki) (6).

Gene symbol	***Description***	***Log2 of the smallest half-life change between dY and the WT***	***Log2 of the smallest steady-state change between dY and the WT***	***S. aureus NCTC8325-4 log2 (fold_change)***
		RNase-Y mutant in S. aureus N315 strain (Khemici et al. 2015)		***Δqrp/yheAΔymcAΔylbF mutant strain***
**Virulence**				
spa	protein A	-0,5	-3,4	4,1
capA	Capsular polysaccharide biosynthesis protein	0,0	0,9	-2,0
capB	Capsular polysaccharide biosynthesis protein Cap5B	0,0	0,6	-2,1
capC	Capsular polysaccharide biosynthesis protein Cap8C	0,0	0,8	-2,2
capD	Capsular polysaccharide biosynthesis protein Cap5D	0,0	0,5	-2,0
capE	Capsular polysaccharide biosynthesis protein Cap5E	-0,1	0,5	-1,5
capF	Capsular polysaccharide biosynthesis protein Cap8F	0,0	0,3	-1,4
capG	UDP-N-acetylglucosamine 2-epimerase	-0,2	0,5	-1,2
capH	Capsular polysaccharide biosynthesis protein O-acetyl transferase Cap5H	0,0	0,5	-1,1
capI	Capsular polysaccharide biosynthesis protein Cap5I	0,0	1,0	-1,1
capJ	Capsular polysaccharide biosynthesis protein Cap5J	0,0	1,0	-1,4
capL	Cap5L protein/glycosyltransferase	-1,1	0,4	-0,6
capP	UDP-N-acetylglucosamine 2-epimerase	-0,9	0,3	0,6
saeS	Histidine protein kinase SaeS	-0,1	0,0	-0,6
saeR	Response regulator	0,0	0,0	-0,7
capK	Capsular polysaccharide biosynthesis protein Cap5K	0,0	1,8	-1,2
rot	Virulence factor regulator protein	0,0	0,0	-0,6
splF	Serine protease SplF	-0,1	0,1	-8,4
splD	Serine protease SplD	0,0	0,0	-8,5
splC	Serine protease SplC	0,0	0,0	-7,8
splB	Serine protease SplB	0,0	0,0	-7,8
splA	Serine protease SplA	-0,6	0,0	-7,9
lukD	Leukotoxin LukD	0,0	1,4	-3,2
lukE	Leukotoxin LukE	0,0	0,4	-3,8

sigB	RNA polymerase sigma factor SigB	0,0	0,0	-0,6
rpsM	Phenol-soluble modulins (PSMs)	-0,4	0,0	-2,6
hlgA	Alpha-hemolysin	0,0	0,0	-2,5
hlgC	Gamma-hemolysin component C	0,1	0,0	-4,0
hlgB	Gamma-hemolysin component B	0,2	0,0	-3,8
Virulence and Biofilm ica-independent				
sarA	Accessory regulator A	1,9	1,6	0,3
agrA	Accessory gene regulator protein A	1,1	1,9	-1,5
agrC	Accessory gene regulator protein C	1,3	2,1	-1,2
hld	Delta-hemolysin	1,2	1,4	-3,6
agrD	Agr autoinducing peptide	0,3	1,0	-1,1
agrB	Accessory gene regulator protein B	0,8	2,4	-0,9
Adhesion Proteins				
sdrC	Fibrinogen-binding protein SdrC	0,0	0,0	-2,0
sdrD	Fibrinogen-binding protein SdrD	0,0	0,0	1,3
clfA	Clumping factor	0,0	0,3	-0,8
map	MHC class II analog protein	0,8	0,0	-1,6
Adhesion Proteins and Biofilm ica-independent				
atl	Bifunctional autolysin	0,5	0,3	0,9
fnbB	Fibronectin binding protein B	0,0	0,0	0,8
Biofilm ica-independent				
tagA	Teichoic acid biosynthesis protein	0,4	0,0	-0,8
tagB	Teichoic acid biosynthesis protein TagB	0,2	0,5	-0,8
arlS	Signal transduction histidine-protein kinase ArlS	0,5	0,5	-1,0
sbi	Immunoglobulin G-binding protein Sbi	0,3	0,0	-0,9
Stabilised ORFs with an associated RNas- Y cleavage site				
dnaX	DNA polymerase III subunits gamma and tau	1,2	0,9	0,7
rplY	50S ribosomal protein L25/general stress protein Ctc	3,1	2,5	1,2
rplA	50S ribosomal protein L1	2,1	1,1	1,3
Gap (gapA)	Glyceraldehyde-3-phosphate dehydrogenase	1,1	0,1	0,0
cggR	Glycolytic operon regulator	NR	NR	0,9

fabD	Malonyl CoA-acyl carrier protein transacylase	1,1	1,5	0,8
rpsB	30S ribosomal protein S2	3,3	4,4	ND
rpsO	30S ribosomal protein S15	1,6	1,0	1,2
glnR	Glutamine synthetase repressor	1,5	0,2	1,2
glyS	Glycyl-tRNA synthetase	1,7	1,0	0,8
rpsU	30S ribosomal protein S21	3,2	3,3	ND
dnaK	Molecular chaperone DnaK	2,3	2,0	0,7
grpE	Heat shock protein GrpE	3,3	2,6	0,8
hrcA	Heat-inducible transcription repressor HrcA	3,8	2,9	0,6
valS	Valyl-tRNA synthetase	1,3	0,8	0,6
SA1734 (pncA)	Pyrazinamidase/nicotinamidase	1,1	1,3	1,0
SA1735 (ppaC)	Manganese-dependent inorganic pyrophosphatase	1,1	0,8	0,5
RNase-Y cleavage sites associated with degradosome ORFs				
SA0941 (rpoY)	Hypothetical protein	0,0	0,6	ND
rnjA	RNase J1	0,2	0,0	0,4
rnjB	Hypothetical protein	0,3	0,1	ND
pfkA	6-phosphofructokinase	0,0	0,0	0,9
cvfA	Ribonuclease Y	1,0	-6,2	0,0
T-box riboswitches cleaved by RNase-Y				
valS	Valyl-tRNA synthetase	1,3	0,8	0,6
leuS	Leucyl-tRNA synthetase	0,3	0,5	0,8
serS	Seryl-tRNA synthetase	-0,2	0,0	0,7
glyS	Glycyl-tRNA synthetase	1,7	1,0	0,8
thrS	Threonyl-tRNA synthetase	0,2	0,0	0,4

ND: Not determined. NR: Not registered. The genes were compared with the repository of the *Staphylococcus aureus* research & annotation community (AureoWiki) (Fuchs *et al.* 2018) (6).

References

1. Bæk KT, Frees D, Renzoni A, Barras C, Rodriguez N, Manzano C, Kelley WL. 2013. Genetic Variation in the Staphylococcus aureus 8325 Strain Lineage Revealed by Whole-Genome Sequencing. PLOS ONE 8:e77122.

2. Monk IR, Tree JJ, Howden BP, Stinear TP, Foster TJ. 2015. Complete Bypass of Restriction Systems for Major Staphylococcus aureus Lineages. mBio 6:e00308-15.

3. Charpentier E, Anton AI, Barry P, Alfonso B, Fang Y, Novick RP. 2004. Novel cassette-based shuttle vector system for gram-positive bacteria. Appl Environ Microbiol 70:6076-85.

4. Arnaud M, Chastanet A, Debarbouille M. 2004. New vector for efficient allelic replacement in naturally nontransformable, low-GC-content, gram-positive bacteria. Appl Environ Microbiol 70:6887-91.

5. Khemici V, Prados J, Linder P, Redder P. Decay-Initiating Endoribonucleolytic Cleavage by RNase Y Is Kept under Tight Control via Sequence Preference and Sub-cellular Localisation. PLOS Genetics. 2015;11(10):e1005577.

6. Fuchs S, Mehlan H, Bernhardt J, Hennig A, Michalik S, Surmann K, et al. AureoWiki The repository of the Staphylococcus aureus research and annotation community. Int J Med Microbiol. 2018;308(6):558-68.
